# Sexual Crossing, Chromosome-Level Genome Sequences, and Comparative Genomic Analyses for the Medicinal Mushroom *Taiwanofungus Camphoratus* (Syn. *Antrodia Cinnamomea*, *Antrodia Camphorata)*

**DOI:** 10.1128/spectrum.02032-21

**Published:** 2022-02-23

**Authors:** Chia-Ling Chen, Wan-Chen Li, Yu-Chien Chuang, Hou-Cheng Liu, Chien-Hao Huang, Ko-Yun Lo, Chung-Yu Chen, Fang-Mo Chang, Guo-An Chang, Yu-Ling Lin, Wen-Der Yang, Ching-Hua Su, Tsung-Ming Yeh, Ting-Fang Wang

**Affiliations:** a Institute of Molecular Biology, Academia Sinica, Taipei, Taiwan; b Shen Nong Fungal Biotechnology Co. Ltd., Taoyuan City, Taiwan; c School of Dentistry, College of Oral Medicine, Taipei Medical University, Taipei, Taiwan; d KFK Biotech Co. Ltd., Kaohsiung, Taiwan; e HIMA Foundation, Taipei, Taiwan; f Department of Microbiology and Immunology, Taipei Medical University, Taipei, Taiwan; MRC Centre Medical Mycology at the University of Exeter

**Keywords:** *Antrodia cinnamomea*, genome-wide annotation, near-complete genome sequences, mushroom

## Abstract

*Taiwanofungus camphoratus* mushrooms are a complementary and alternative medicine for hangovers, cancer, hypertension, obesity, diabetes, and inflammation. Though *Taiwanofungus camphoratus* has attracted considerable biotechnological and pharmacological attention, neither classical genetic nor genomic approaches have been properly established for it. We isolated four sexually competent monokaryons from two *T. camphoratus* dikaryons used for the commercial cultivation of orange-red (HC1) and milky-white (SN1) mushrooms, respectively. We also sequenced, annotated, and comparatively analyzed high-quality and chromosome-level genome sequences of these four monokaryons. These genomic resources represent a valuable basis for understanding the biology, evolution, and secondary metabolite biosynthesis of this economically important mushrooms. We demonstrate that *T. camphoratus* has a tetrapolar mating system and that HC1 and SN1 represent two intraspecies isolates displaying karyotypic variation. Compared with several edible mushroom model organisms, *T. camphoratus* underwent a significant contraction in the gene family and individual gene numbers, most notably for plant, fungal, and bacterial cell-wall-degrading enzymes, explaining why *T. camphoratus* mushrooms are rare in natural environments, are difficult and time-consuming to artificially cultivate, and are susceptible to fungal and bacterial infections. Our results lay the foundation for an in-depth *T. camphoratus* study, including precise genetic manipulation, improvements to mushroom fruiting, and synthetic biology applications for producing natural medicinal products.

**IMPORTANCE** *Taiwanofungus camphoratus* (Tc) is a basidiomycete fungus that causes brown heart rot of the aromatic tree Cinnamomum kanehirae. The Tc fruiting bodies have been used to treat hangovers, abdominal pain, diarrhea, hypertension, and other diseases first by aboriginal Taiwanese and later by people in many countries. To establish classical genetic and genomic approaches for this economically important medicinal mushroom, we first isolated and characterized four sexually competent monokaryons from two dikaryons wildly used for commercial production of Tc mushrooms. We applied PacBio single molecule, real-time sequencing technology to determine the near-completed genome sequences of four monokaryons. These telomere-to-telomere and gapless haploid genome sequences reveal all genomic variants needed to be studied and discovered, including centromeres, telomeres, retrotransposons, mating type loci, biosynthetic, and metabolic gene clusters. Substantial interspecies diversities are also discovered between Tc and several other mushroom model organisms, including Agrocybe aegerita, Coprinopsis cinerea, and Schizophyllum commune, and Ganoderma lucidum.

## INTRODUCTION

*Taiwanofungus camphoratus* (Basidiomycota, Polyporales) (1), known as Bull camphor fungus (“Niu-Chang-Chih”), is a medicinal mushroom with a high content (>30% wet weight) of naturally produced chemicals. It was also known as *Ganoderma camphoratum* (2), *Antrodia cinnamomea* (*Ac*) (3), and *Antrodia camphorata* (4). The percentage of methanolic extracts from *T. camphoratus* mushrooms is 10 times greater than for Ganoderma lucidum fruiting bodies (FBs) (2). With an annual market of over $100 million, *T. camphoratus* has received considerable attention in the pharmacology and biotechnology sectors (5, 6). The first *T. camphoratus* genome draft was determined by next-generation sequencing (NGS) technology using genomic DNA isolated from s27, a primary mycelium (monokaryon) (7). However, due to the technical limitations of NGS technology (8, 9), as well as high copy numbers of long terminal repeat (LTR) retrotransposons (RTs) in the *T. camphoratus* genome, the s27 genome draft (32.15-Mb, 360 scaffolds) not only is far from near-completed assembly but also contains many assembly and annotation errors (see below). Genome-wide gene prediction and transcriptomic analyses were performed using total RNAs isolated from mycelia of two monokaryons (s27 and s32), a dikaryotic mycelium (AM), and a wood-grown FB (AT). Neither AM nor AT were derived from s27 or s32.

Sexual crossing methods for classical genetic studies of *T. camphoratus* also have not yet been established. In this study, we isolated and phenotypically characterized four sexually competent *T. camphoratus* monokaryons from two commercial dikaryons, established a sexual crossing method under laboratory conditions, determined their chromosome-level genome sequences, and performed comprehensive genome-wide annotations. Comparative genomic and transcriptomic analyses were carried out for different developmental stages, as well as between several other edible mushrooms. Our findings provide many new insights into this valuable medicinal mushroom.

## RESULTS

### Establishment of a sexual crossing system for genetic and genomic analyses.

**FIG 1:**
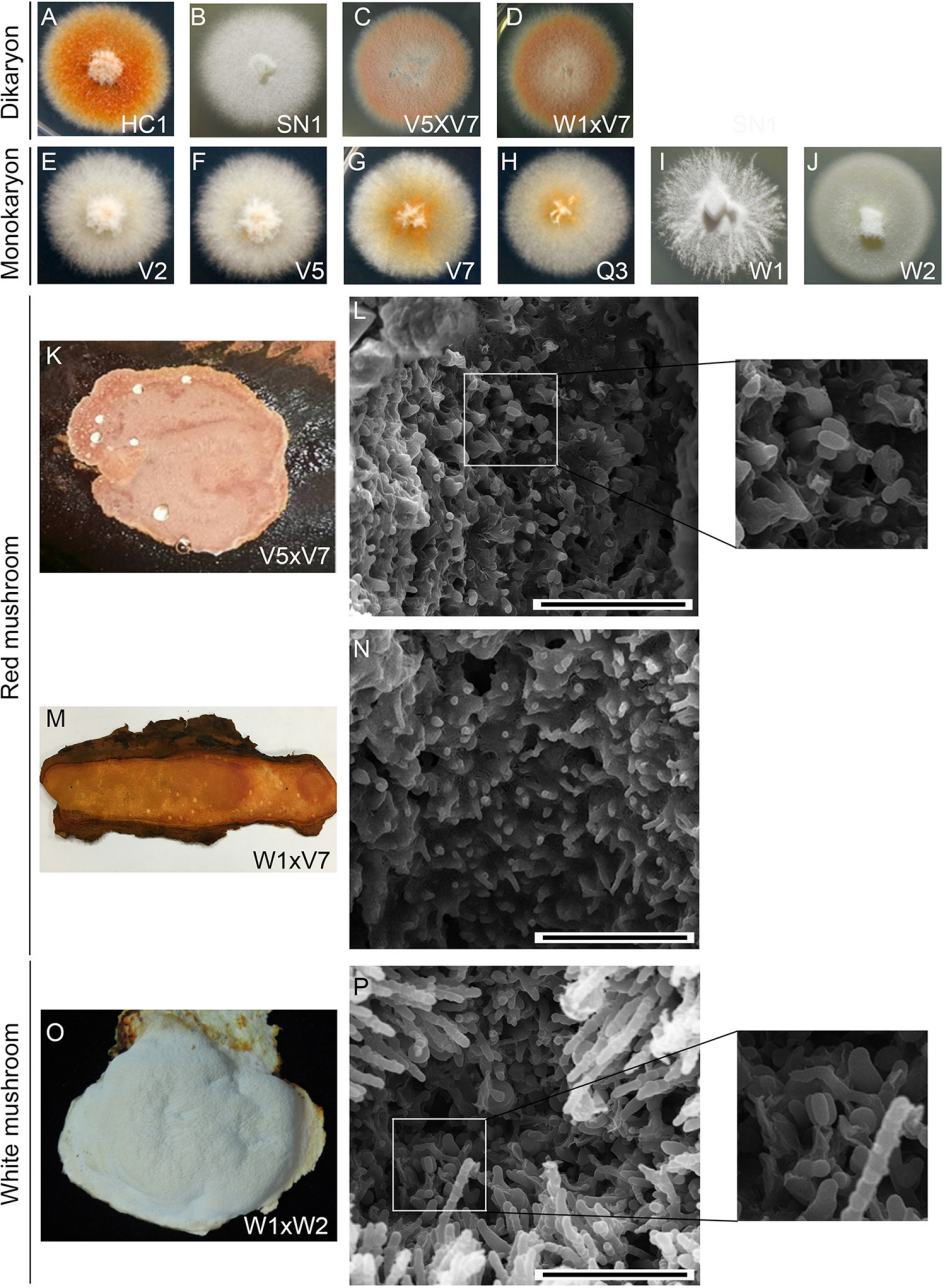
Morphological characterization of *T. camphoratus* vegetative mycelia and fruiting bodies. (A to J) Colony morphology and color of vegetative dikaryotic and monokaryotic cultures. (K to P) Cryo-scanning electron microscopy images of the surfaces of three representative *T. camphoratus* mushrooms. Only W1×W2 and V5×V7 (but not W1×V7) mushrooms show a few basidia with four basidiospores. The scale bar = 0.04 mm.

HC1 is a dikaryon derived from an orange-red-colored mushroom isolated from a farm at Nantou, Taiwan (Fig. 1A). SN1, another dikaryon, was isolated from a creamy-white mushroom by Shen Nong Fungal Biotechnology Co. Ltd. (Taoyuan, Taiwan) (Fig. 1B). V2, V5, V7, and Q3 are the monokaryons germinated from arthrospores taken from HC1 vegetative mycelia, respectively (Fig. 1E to H). Arthrospores are asexual spores that derived from individual cells disarticulate from the growing *T. camphoratus* hyphae in vegetative cultures and developing mushrooms. W1 and W2 are the monokaryons germinated from arthrospores taken from SN1 vegetative mycelia (Fig. 1I and J; Table S1).

The mating compatibility of these six monokaryons was determined by mixing combinations of their mycelia on Malt Extract Agar plates. We found that W1, V2, and V5 could mate with W2, V7, or Q3 to form dikaryotic mycelia with clamp connections, respectively. Interestingly, W2 was sexually incompatible with V7 and Q3 (Table 1). Judging from their respective origins and their mating compatibility, V2 and V7 are likely isogenic to V5 and Q3, respectively (Table 1).

**TABLE 1:** Sexual mating test^*a*^

Monokaryon	W1	W2	V2	V5	V7	Q3
W1	−	+	−	−	+	+
W2		−	+	+	−	−
V2			−	−	+	+
V5				−	+	+
V7					−	−
Q3						−

a The ability of two monokaryons to mate and form dikaryotic hyphae with clamp connections is indicated as “+”and "−".

Three new dikaryons (W1×W2, V5×V7, and W1×V7) were generated via sexual mating of the corresponding monokaryons. All three of these dikaryotic mycelia could fruit on aged heartwood of the bull camphor tree (*Cinnamomum kanehirae*; *Ck*) (10). Like SN1, the W1xW2 dikaryon fruited and formed creamy-white mushrooms, whereas both the V5×V7 and W1×V7 dikaryons formed orange-red mushrooms like those of HC1. We also performed an imaging analysis using cryogenic scanning electron microscopy (Cryo-SEM) to show that the mushrooms of W1×W2 (*n* = 5) and V5×V7 (*n* = 4) formed basidia and basidiospores (Fig. 1P and L). In contrast, no basidia or basidiospores were ever observed for W1×V7 mushrooms (*n* = 6) (Fig. 1N).

### Complete genome sequencing, *de novo* assembly, and annotation.

We determined the high-quality and nearly complete genome sequences of W1, W2, V5, and V7 using both PacBio and Illumina platforms (Table 2 and Tables S2 to S6) (8, 9). The error rates for our four haploid genome drafts are extremely low. First, high PacBio sequencing coverage indicated no sequence ambiguities or unidentified bases (Ns) (8). Second, the four haploid genome drafts each encompass a circular mitochondrial genome (mitogenome). There is only one nucleotide difference between the mitogenome sequences of W1 (115,207 basepairs) and W2 (115, 206 bp) or between V5 (107,517 bp) and V7 (107,518 bp), respectively (Table S6). Therefore, even without error correction on NGS sequencing reads, error rates for our PacBio assembly results are ≤8.68 × 10^−6^. Compared with the mitogenomes of W1 and W2, those of V5 and V7 display two internal deletions (Fig. S1 and S2). The s27 mitogenome (115,204 bp) is almost completely identical to those of W1 and W2 (7). Mitogenomes are inherited exclusively from the mother (i.e., maternal inheritance) during sexual reproduction of most eukaryotes, so we inferred that s27, W1, and W2 share the same maternal lineage.

**TABLE 2:** Summary of the annotated genes of four nearly complete *T. camphoratus*/*A. cinnamomea* (*Ac*) genome sequences

Strain	W1	W2	V5	V7
Locus_tag	AcW1	AcW2	AcV5	AcV7
Genome (bps)	33,373,109	33,571,907	32,855,596	33,286,329
BUSCO genome metrics	96.1 %	95.8 %	95.9 %	96.2 %
BUSCO protein metrics	94.6 %	96.6 %	95.9 %	96.2 %
Distinct protein encoding genes	10,247	10,273	10,401	10,308
Total proteins	14,019	13,965	14,141	14,055
tRNAs	75	73	72	87
LncRNAs	3,835	3,808	3,530	4,007
Transcription factors	427	438	442	418
Proteins with predicted signal peptide	650	648	651	639
Carbohydrate-Active enZymes (CAZymes)				
Auxiliary activity (AA)	25	27	28	25
Carbohydrate-binding modules (CBM)	3	5	5	6
Carbohydrate esterases (CE)	5	7	7	7
Glycoside hydrolases (GH)	105	112	109	111
Glycosyl transferases (GT)	49	48	51	49
Polysaccharide lyases (PL)	3	3	3	2
CAZyme gene clusters (CAZ-GCs)	2	5	3	6
Secondary metabolism (SM) biosynthetic enzymes				
NRPS	2	2	2	2
PKS/NRPS-like protein	8	7	7	7
Hybrid PKS-NRPS	1	1	1	1
NRPS-like protein	8	6	5	7
Type I Iterative PKS	4	4	5	4
CYP	74	75	79	77
SM biosynthetic gene clusters (SM-BGCs)	26	25	26	26

For genome-wide gene prediction and transcriptomic analyses, we applied Illumina NGS paired-end sequencing technology to determine RNA transcripts from mycelia of six monokaryons (W1, W2, V2, V5, V7, and Q3), two dikaryons (W1×W2 and V5×V7), as well as from three FBs (W1×W2, V5×V7 and W1×V7) (Table S7). We identified 10,247, 10,273, 10,401, and 10,308 distinct protein-coding gene models (13,965 to 14,141 protein-coding transcripts), respectively, in the four haploid genomes of W1, W2, V5, and V7 (Table 2 and Data Set 2 to 5). We also identified and annotated 3,835, 3,808, 3,530, and 4,007 long non-coding RNA (lncRNAs) genes, respectively, in these four haploid genomes (Table 2 and Data Set 6 to 9).

We found that the BUSCO protein metric for the s27 annotation data set (7) reflected only 90.9% completeness (Table S6). Because the s27 and s32 data sets are not publicly accessible, we reannotated the NGS-based genome draft using “Funannotate” against all of the NGS-based RNA sequencing data sets generated in our study (Table S7). Our new annotation results reveal many spurious protein-encoding genes (*n* = 11,809) and a new BUSCO protein metric of 96.5% completeness (Table S6), indicating that the s27 genome draft harbors many duplicated genome sequences due to improper assembly or annotation (7).

### *T. camphoratus* genomes harbor large numbers of retrotransposons.

**FIG 2:**
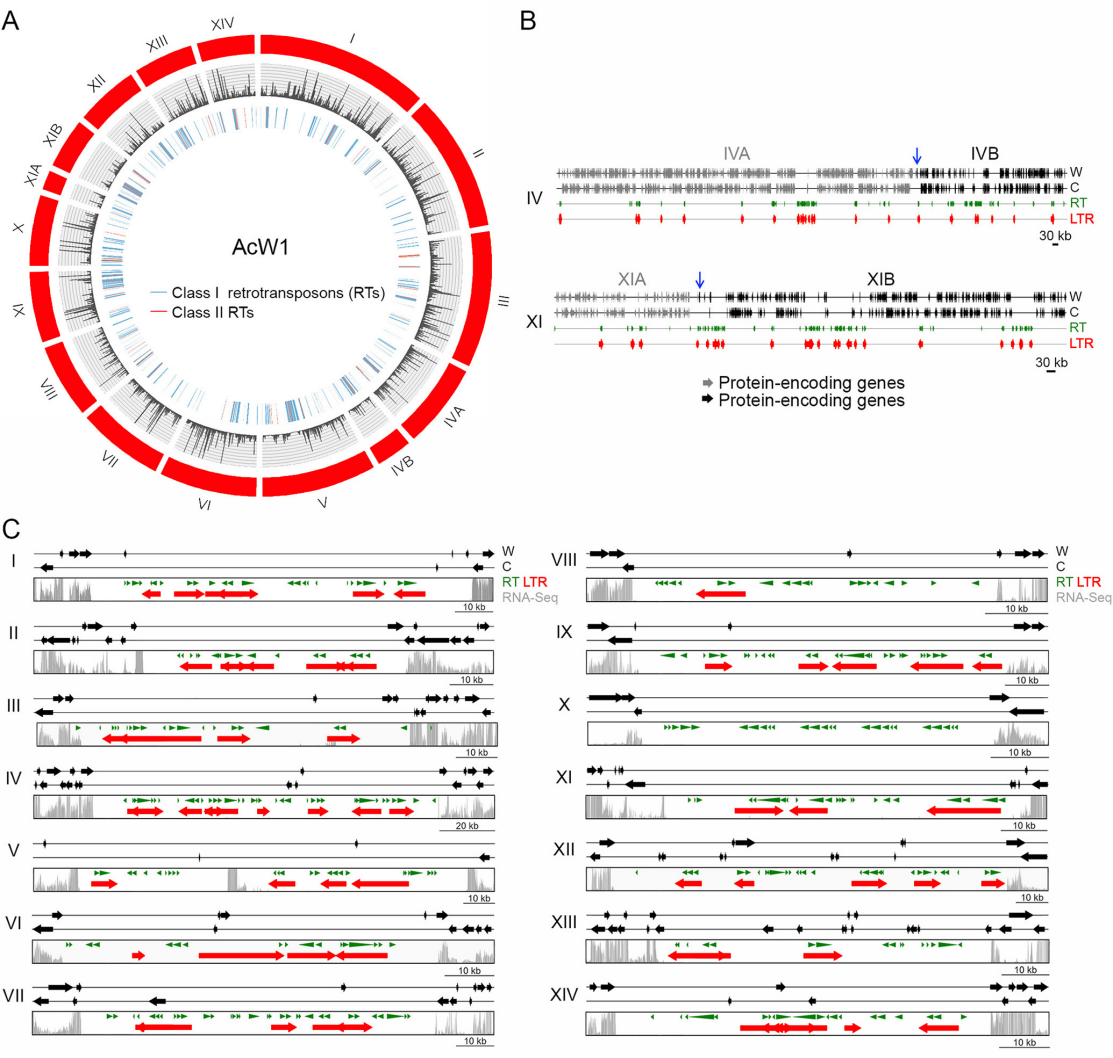
The 14 W1 chromosomes and their centromeres. (A) Visualization of the 14 W1 chromosomes by using CIRCOS (http://circos.ca). The outer circle indicates all chromosomes and superscaffolds of W1 (in red). The middle circle is a plot of RNA-seq read depth. Class I RTs (in blue) and class II RTs (in red) are shown in the inner traces. (B) Schematic illustrating the overlapping regions between ChIVA (in gray) and ChIVB (in black), as well as between ChXIA (in gray) and ChXIB (in black), respectively. The overlapping regions are indicated by blue arrows. (C) Schematic illustrating the 14 putative W1 centromeric regions. These intervals were defined as the longest ORF-free regions on the respective chromosomes and mostly contain RTs. RNA sequencing analysis revealed that the identified centromeric regions also presented reduced levels of transcriptional activity relative to flanking non-centromeric regions. The gray bars indicate RNA-seq read depth. RTs predicted by the RepeatMasker search program (http://www.repeatmasker.org/) (8) and the LTR-finder program (http://tlife.fudan.edu.cn/tlife/ltr_finder/) (12) are indicated in green and red, respectively. The locations of all 14 W1 centromeres are listed in Table S8.

The four haploid *T. camphoratus* genomes each consists of 4.7% to 6.1% repetitive sequences, mainly class I and class II RTs. We identified a massive burst-like expansion of Gypsy, a class I RT with LTRs. The overall copy number of Gypsy-LTRs ranged from 1,157 in V5 to 1,259 in V7 (Table S6). The majority of RTs are scattered throughout the four genomes, but some are preferentially clustered in specific chromosomal regions, such as in the mating-type loci and centromeres (see below). As reported previously (11), the RTs in fungal genomes influence genome-wide gene transcription, displaying stronger repression of their nearby protein-coding genes (Fig. 2A).

### The haploid *T. camphoratus* genome has 14 nuclear chromosomes.

The genome assembly data set for W1 contains 16 chromosome-scale superscaffolds (Fig. 2A and Table S2). From the respective nucleotide sequences and the results of pulsed-field gel electrophoresis (PFGE) and Southern hybridization (Fig. S3), we conclude that W1 contains 14 full-length nuclear chromosomes, the termini of which all display typical telomeric sequences (i.e., TTAGGG at 3′-termini and the reverse complement CCCTAA at 5′-termini) (Table S2). We have denoted these 14 telomere-to-telomere W1 chromosomes with Roman numerals (i.e., ChI to ChXIV), from largest to smallest (Fig. 2A). The putative centromeric loci were identified the longest regions of each W1 chromosome lacking an open-reading frame (ORF) and mostly containing RTs or their remnants (Fig. 2C and Table S8). The same method was used previously to define the centromeric loci of the *Crytococcus amylolentus* genome (12). The 14 assembled W1 chromosomes are syntenic to all supercaffolds in the other three *T. camphoratus* haploid genomes. For example, the W2 genome draft contains 18 superscaffolds (Fig. S4). Four W2 chromosomes (ChII, ChIV, ChIX, and ChXI) were each divided into two superscaffolds, respectively. All corresponding break-points in these four chromosomes are located in RT-enriched chromosomal regions.

### Karotype heterogeneity.

**FIG 3:**
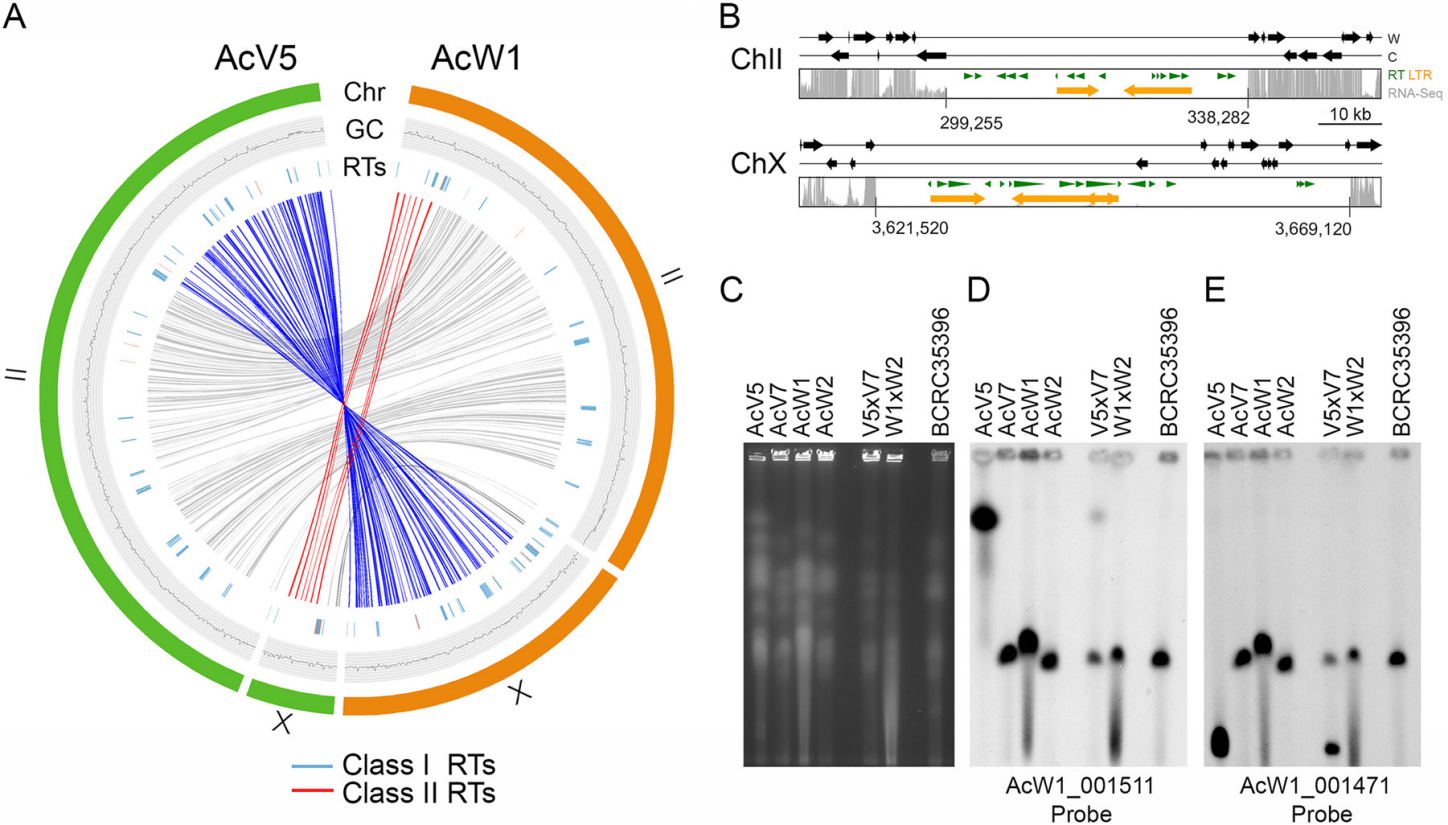
Chromosomal translocations in V5. (A) Collinearity relationships are depicted in red, blue, or gray in the inner circle, respectively. The outer circles represent W1-ChII and W1-ChX (in orange), as well as V5-ChII and V5-ChX (in green). The GC contents (window size 5,000 bp) of the two chromosomes are shown in the middle traces. Class I RTs (in blue) and class II RT (in red) are shown in the inner traces. (B) Schematic illustrating the putative centromeric regions on V5-ChII and V5-ChX. The gray bars indicate RNA-seq read depth. RTs predicted by the RepeatMasker search program (http://www.repeatmasker.org/) (8) and the LTR-finder program (http://tlife.fudan.edu.cn/tlife/ltr_finder/) (12) are indicated in green and orange, respectively. The locations of two V5 centromeres are indicated. (C) PFGE was applied to separate the chromosomes of V5, V7, W1, W2, V5×V7, W1×W2, and BCRC35396. (D to E) Southern hybridization with two DNA probes, as indicated. These results provide additional evidence of our high-quality genome assembly for W1 and V5.

Fungal genomes often exhibit higher rates of RT-mediated gross chromosomal rearrangement (GCR) during evolution than those of other eukaryotes (13). The *T. camphoratus* genomes also presented this property. The most profound example of that phenomenon is the second and 10th W1 chromosomes (W1-ChII, 3,401,759 bp; W1-ChX, 1,738,909 bp) (Fig. 3A, right panel) underwent unequal and reciprocal rearrangement in V5, resulting in this latter having a much longer second chromosome (V5-ChII; 4,545,385 bp) and a shorter tenth chromosome (V5-ChX; 488,068 bp) (Fig. 3A, left panel). Notably, the four corresponding GCR break-points contain or are close to RTs. We applied PFGE karyotyping to separate intact chromosomal-sized DNA from four monokaryotic mycelia (W1, W2, V5, and V7) and three dikaryotic mycelia (V5×V7, W1×W2, and BCRC35396). BCRC35396 is the holotype of *T. camphoratus* (3). BCRC35396 is no longer used for mushroom cultivation because it underwent senescence (i.e., cellular aging) and became incompetent for fruiting (unpublished results by CCC, TFW, CYC, and ZMY). Southern hybridization with two different DNA probes revealed that length polymorphisms for the second and 10th chromosomes only exist in V5 and V5×V7, and not in W1, W2, V7, W1XW2, or BR35396 (Fig. 3C to E).

### Copy number variability among 45S ribosomal DNA arrays.

Using PFGE karyotyping and Southern hybridization with a ChVIII DNA probe (i.e., the AcW1 009562 gene as an internal control for Southern hybridization) (Fig. S5A) and a ChVII DNA probe (i.e., the AcW1 008299 gene) (Fig. S5B), we found that ChVII in V7 is much shorter than the corresponding chromosomes in V2, V5, Q3, W1, and W2 (Fig. S5B, right panel). The terminus of the right arm of ChVII harbors the tandem “head-to-tail” repeats of the 45S rDNA locus. Each repeat contains an 18S-5.8S-28S gene cluster and a non-transcribed intergenic spacer (IGS) (Fig. S5C). Because V7 and Q3 are derived from two “isogenic” HC1 arthrospores, we inferred that V7 (but not Q3) might have undergone a loss in rDNA copy number. Two lines of evidence support that notion. First, the relative Southern hybridization signal intensity of 18S rDNA in V7 was much weaker than for that of the other five monokaryons (Fig. S5A, middle panel). Second, the relative mapping coverage of Illumina MiSeq reads for rDNA compared to that of single-copy genes is much lower in V7 (21.4-fold) than in Q3 (38.1-fold), V2 (52.5-fold), V5 (48.1-fold), W1 (37.6-fold), and W2 (39.6-fold), respectively (Fig. S5D).

According to the rDNA theory of cellular aging (14), genome instability is the primary aging signal and rDNA is more sensitive to age-related damage than other regions of the genome. Loss of rDNA copies is associated with hypersensitivity to DNA damage agents in mammalian cells. For instance, low rDNA copy number reduces the number of ribosomes in Saccharomyces cerevisiae, leading to higher sensitivity to cycloheximide, an inhibitor of protein synthesis (15). We exposed six monokaryotic mycelia (W1, W2, V2, V5, V7, and Q3) to the DNA damage agent methyl methanesulfonate (MMS), and then compared their vegetative growth rates on Malt Extract Agar plates. We found that V7 was slightly more sensitive to 0.02% MMS than V5, Q3, W1, and W2 during vegetative growth (Fig. S5E). However, the reduced rDNA copy number of V7 does not appear to affect sexual mating or mushroom development because V7 could mate with W1 or V5 to form dikaryotic mycelia with clamp structures (Table 1). Both V5×V7 and W1×V7 fruited on aged *Ck* basswood (Fig. 1).

### *T. camphoratus* has a tetrapolar mating-type system.

Sexual reproduction in basidiomycete fungi involves two distinct mating features, i.e., homothallism (self-fertilization) and heterothallism (whereby two compatible partners are required to produce sexual spores). Heterothallism can be further classified as bipolar or tetrapolar based on genetic linkage of the *matA* (*HD*) mating-type locus and the *matB* (*P/R*) mating-type locus. The *matA* locus contains *HD1* and *HD2* genes, whereas the *matB* locus mainly includes pheromone G-protein-coupled receptor genes (*ste3/pra*) and a-factor mating pheromone genes (*mfa*) (16, 17). To identify these mating-type genes (i.e., *HD1*, *HD2*, *ste3*, and *mfa*) in *T. camphoratus*, we subjected all RNA-seq data set (Table S7) generated in this study, as well as that from the s27 monokaryon (7), to *de novo* transcriptome assembly using the “Trinity” software tool (supplemental appendix, “Materials and Methods”). The resulting transcriptome revealed a total of 48 mating-type genes, including five *HD1* genes, five *HD2* genes, 16 *mfa* genes, and 22 *ste3* genes (Table S9 and Fig. S6 to S9). The s27, W1, W2, and V5 genomes each possesses different *HD1* and *HD2* genes, but the *HD1* and *HD2* genes of W2 are 100% identical in amino acid sequence to those of V7 (Fig. S10). W1, W2, V5, and V7 share five common *mfa* genes. These four monokaryons also each possesses a specific set of *mfa* and *ste3* genes (Table S9). These results are consistent with those of our sexual mating assay (Table 1), which revealed that W1, V2, and V5 could mate with W2, V7, or Q3, respectively, whereas W2 was sexually incompatible with V7 and Q3.

**FIG 4:**
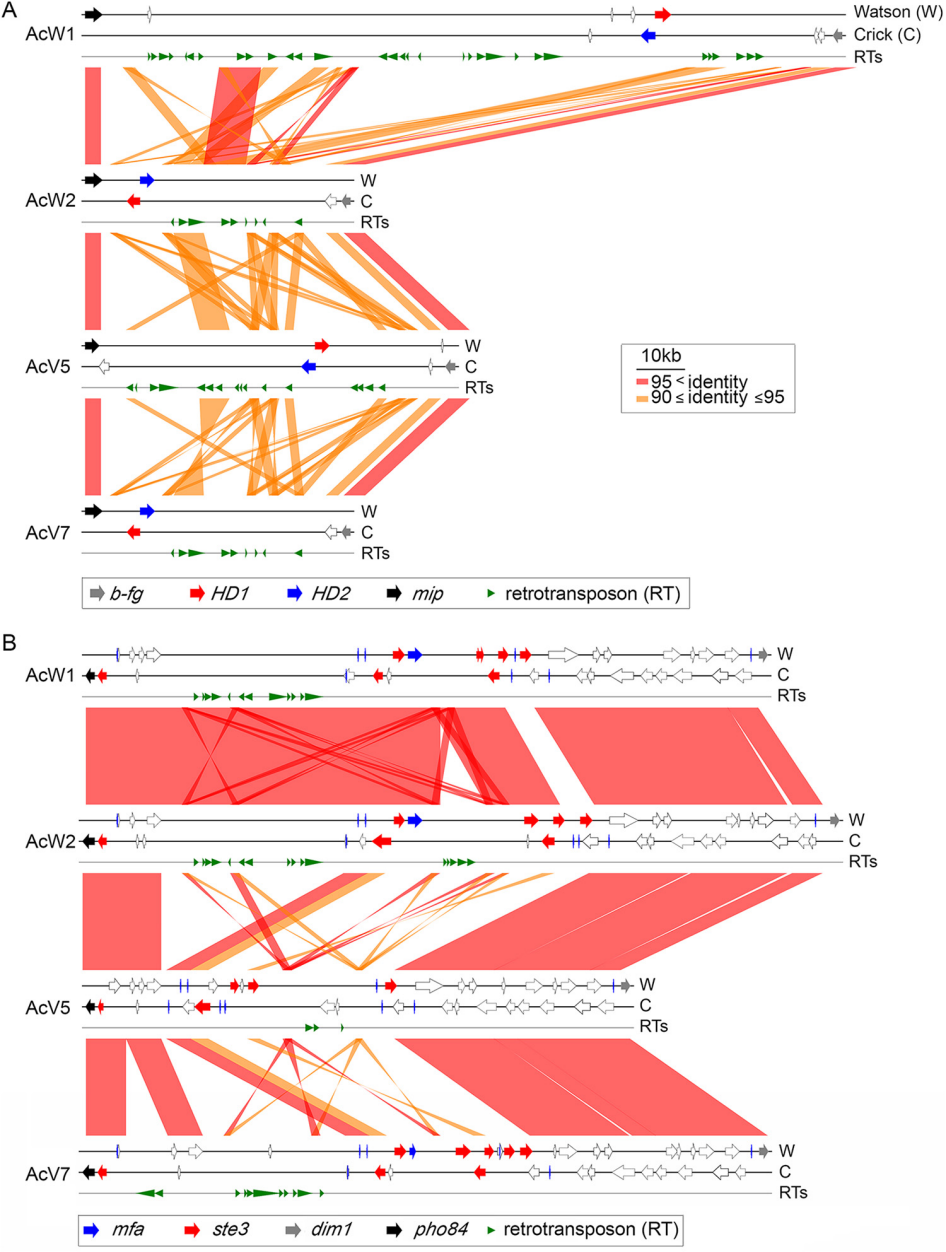
Synteny around the *matA* mating type loci (A) and *matB* mating type loci (B) of W1, W2, V5, and V7. The two DNA strands and retrotransposons are indicated as Watson (W), Crick (C), and RTs, respectively. RTs were predicted by the RepeatMasker search program (http://www.repeatmasker.org/) (8). *HD1*, *HD2*, *b-fg*, *mip1*, *ste3*, *mfa*, *dim1*, and *pho84* are indicated by red, blue, gray, black, red, blue, gray, and black arrows, respectively. Other protein-encoding genes are indicated by open arrows.

As observed for several other basidiomycetes (18, 19), *HD1* and *HD2* are flanked by two evolutionarily-conserved genes, i.e., *b-fg* (a conserved fungal protein gene) and *mip1* (a mitochondrial intermediate peptidase gene). The orientation of the *HD1* and *HD2* genes in W1 and V5 is the opposite of that in W2 and V7 (Fig. 4). All four *matB* loci are flanked by *pho84* (an inorganic phosphate transporter gene) and *dim1* (an essential 18S rRNA demethylase gene). The *matA* and *matB* loci in W1, W2, V5, and V7 each contain different numbers of RTs and chromosomal inversion events, manifesting as length variations. We also performed PFGE karyotyping and Southern hybridization to confirm that *matA* and *matB* are located on two different chromosomes in all six monokaryons (V2, V5, V7, Q3, W1, and W2) (Fig. S11).

We conclude that *T. camphoratus* is a tetrapolar basidiomycete. Our data also clearly demonstrate that HC1 and SN1 represent two wild isolates of the same species, though each displays a range of karyotypic and phenotypic variation.

### Fruiting-related genes.

**FIG 5:**
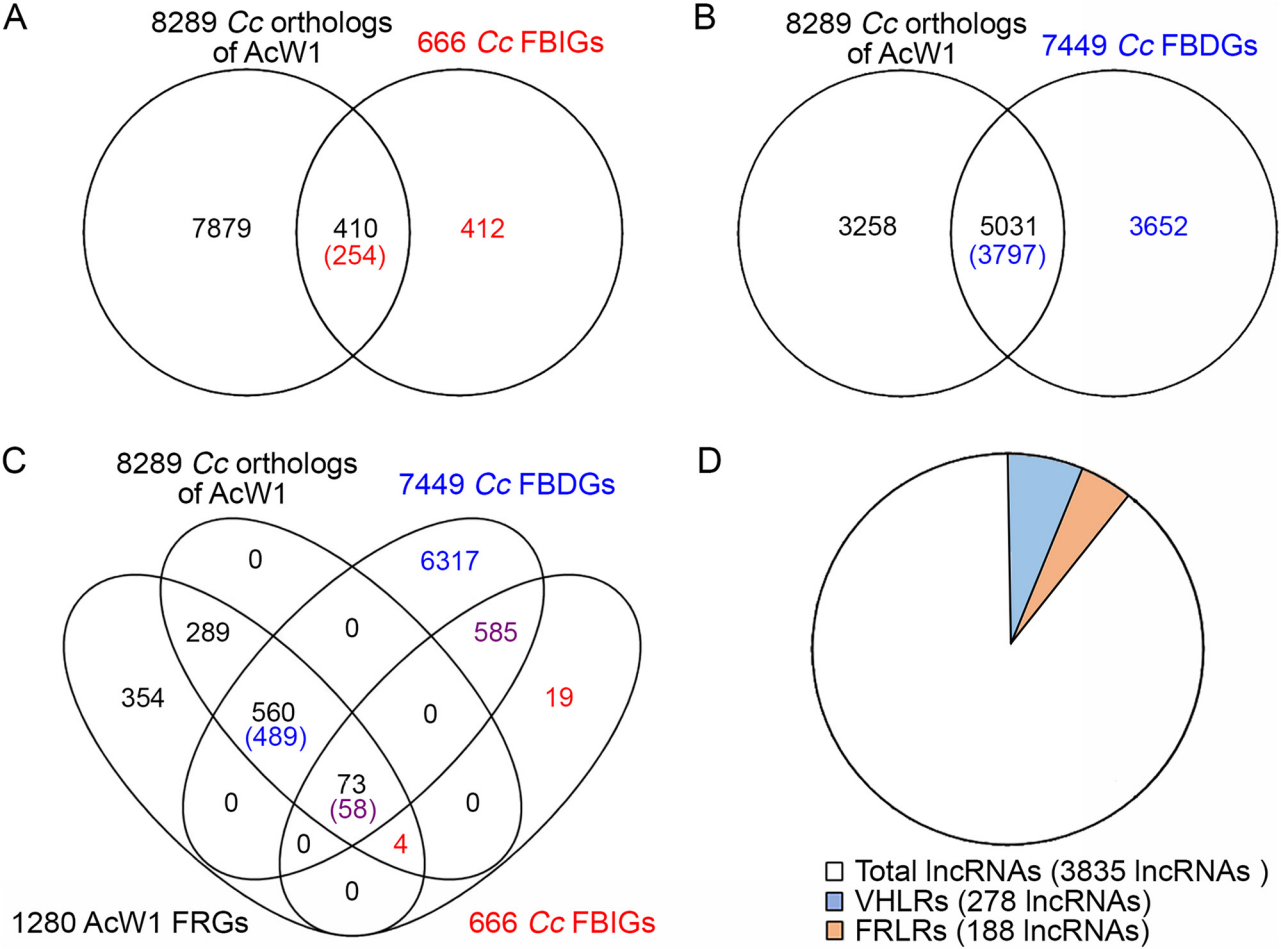
Comparative transcriptomic identification of candidate *T. camphoratus* fruiting-related genes (FRGs) and differentially expressed genes (DEGs) in the orange-red *T. camphoratus* strains. (A, B) VENN diagram reveals that 8,289 *T. camphoratus* W1 protein-encoding genes have at least one ortholog in *C. cinerea*. Of those protein-encoding genes, 254 and 3,797 are highly similar in amino acid sequence to 410 *C. cinerea* FB-initiation genes (*Cc*FBIGs) and 5,031 *C. cinerea* FB development genes (*Cc*FBDGs) (20), respectively. (C) VENN diagram of 1,280 FRGs of *T. camphoratus* W1, of which 73 and 560 are orthologs of 58 *Cc*FBIGs and 489 *Cc*FBDGs, respectively, and a further 354 are *T. camphoratus* specific. Moreover, 289 are neither orthologs of *Cc*FBIGs nor of *Cc*FBDGs. (D) Pie chart of 3,835 lncRNAs in *T. camphoratus* W1. There are 188 fruiting-related lncRNAs (FRLRs) and 278 vegetative hyphae-related lncRNAs (VHLRs).

Mushroom development of the Agaricales or filled mushrooms involves multiple stages. The vegetative mycelia develop sequentially into a hyphal knot, stage 1 primordium, stage 2 primordium, young FB, and finally a mature FB. Although *T. camphoratus* is a polyporus mushroom, the mechanisms of *T. camphoratus* mushroom fruiting are evolutionarily well conserved with respect to those of Agaricales. First, we used BLASTp (E value ≤ e-17) to search for putative *T. camphoratus* genes homologous to the FRGs in various Agaricales mushrooms, including *Agrocybe aegerita*, Coprinopsis cinerea and *Schizophyllum commune*. We identified 24 orthologs of Agaricales fruiting-related genes (FRGs) in the four monokaryon *T. camphoratus* genomes (Table S10). Second, two groups of developmentally-regulated genes in six fungal species have been defined based on their expression profiles during a developmental time-series (20). For example, previous study of *C. cinerea* (*Cc*) revealed that 666 FB-initiation genes (*Cc*FBIGs) display ≥4-fold increases in expression from vegetative mycelia to the stage 1 primordium, and 7,449 FB-development genes (*Cc*FBDGs) show a ≥4-fold increase in expression between any two FB-development time points (20). Our genome-wide BLASTp searches (E ≤ e-5; Data Set 10) identified that *T. camphoratus* possesses at least one homolog of 254 (38.1%) *Cc*FBIGs (Fig. 5A) and 3,797 (50.9.%) *Cc*FBDGs (Fig. 5B and Data Set 11), respectively.

Third, we compared the genome-wide transcriptomic profiles of four monokaryotic vegetative mycelia (W1, W2, V5, and V7), two dikaryotic vegetative mycelia (W1×W2 and V5×V7), as well as three different FBs (W1×W2, V5×V7, and W1×V7) (Fig. S12A). We observed that 1,280 *T. camphoratus* FRGs (Fig. 5C) presented ≥3-fold upregulated expression (*P* < 0.05) in FBs relative to monokaryotic or dikaryotic vegetative mycelia, respectively (Data Set 11). Notably, 73 and 560 of those *T. camphoratus* FRGs are orthologs of *Cc*FBIGs and *Cc*FBDGs, respectively. Furthermore, *T. camphoratus* and Agaricales mushrooms display species-specific fruiting characteristics. For instance, 354 *T. camphoratus* FRGs are *T. camphoratus* specific. Gene ontology (GO) enrichment analyses revealed that the majority of those 354 *T. camphoratus* specific FRGs remain functionally unannotated (Fig. S12B). Moreover, the orthologs of 289 *T. camphoratus* FRGs are neither *Cc*FBIGs nor *Cc*FBDGs (Fig. S12C).

### Genome-wide comparison of the *T. camphoratus* CAZyme repertoire.

We employed the dbCAN2 meta server (http://bcb.unl.edu/dbCAN2) (21) to explore all CAZymes in the four *T. camphoratus* haploid genomes, as well as in four fungal model organisms, *C. cinerea*, *G. lucidum*, *S. commune*, and Pleurotus ostreatus. The dbCAN2 meta server incorporates three different annotation tools, i.e., HMMER, DIAMOND and the Homolog to Peptide Pattern (Hotpep) (21). Next, we applied two different screening methods to search for CAZymes. The less stringent method involved solely using HMMER, whereas the higher stringent method encompassed CAZymes that were identified by at least two annotation tools in dbCAN2. Both screening methods generated similar outputs. All seven fungal genomes we analyzed contained a similar number (∼50) of glycosyl transferases (GT) family genes. In contrast, the four *T. camphoratus* haploid genomes exhibited significant contractions in all five other CAZyme families (GH, AA, CE, PL, and CBM) relative to those of *C. cinerea*, *G. lucidum*, *S. commune*, and *P. ostreatus* (Table S11).

Notably, many CAZyme subfamily genes required for plant and fungal cell wall (PCW or FCW) degradation have been lost from *T. camphoratus* (Table S12), including GH43 (a PCW pectinase and hemicellulase), GH75 and GH76 (PCW chitinases), GH81 (FCW chitinase), GH93 (PCW hemicellulase), GH105 (PCW pectinase; only one gene copy) (22), CE2, CE5, CE8, CE10, CE12, CE14, CE15, CE17, CBM1, CBM5, CBM13, CBM35, CBM63, and CBM67. Notably, CE2, CE5, CE8, CE12, and CE15 are all esterases that facilitate removal of PCW lignin, pectin and the plant polymer cutin by promoting hydrolysis of the carbohydrate ester linkages between glucuronoxylan, acetylxylan, methylpectin, and cutin (23). Our results are consistent with a recent comparative fungal genomic study which revealed that certain CAZyme families (e.g., CBM1, GH6, GH7, and GH10) have disappeared from brown-rot and symbiotic mushrooms (24). *T. camphoratus* also lacks several polysaccharide lyases (PL1, PL2, PL3, PL8, PL9, PL10, PL22, PL26, PL23) that have important roles in the process of host cell penetration by contributing to the degradation of PCW structural polysaccharides (25). Interestingly, we only identified two AA9 genes in *T. camphoratus*, whereas *G. lucidum*, *S. commune*, and *P. ostreatus* have 16, 22, and 29 such genes, respectively. AA9 is a copper-dependent lytic polysaccharide monooxygenase that cleaves cellulose chains via carbon oxidation (26). Thus, our results indicate that *T. camphoratus* is an xylophagous-incompetent or symbiotic brown-rot fungus, perhaps explaining why we found it difficult to cultivate *T. camphoratus* mushrooms on unrotten camphorwood and why wild mushrooms preferentially fruit or are often found on rotten *Ck* tree hearts in nature.

It is also important to note that none of the four *T. camphoratus* genomes possess any lysozyme genes (i.e., GH23-GH26) that target peptidoglycans of bacterial cell walls (22). In contrast, *G. lucidum*, *S. commune*, and *P. ostreatus* possess two, two, and five lysozyme genes, respectively (Table S12). We inferred that *T. camphoratus* might frequently coexist with bacteria. Consistent with this hypothesis, we previously isolated and identified a Gram-negative bacteria, *Burkholderia* sp. WAC0059, from *T. camphoratus* mycelium cultures and FBs (27).

**FIG 6:**
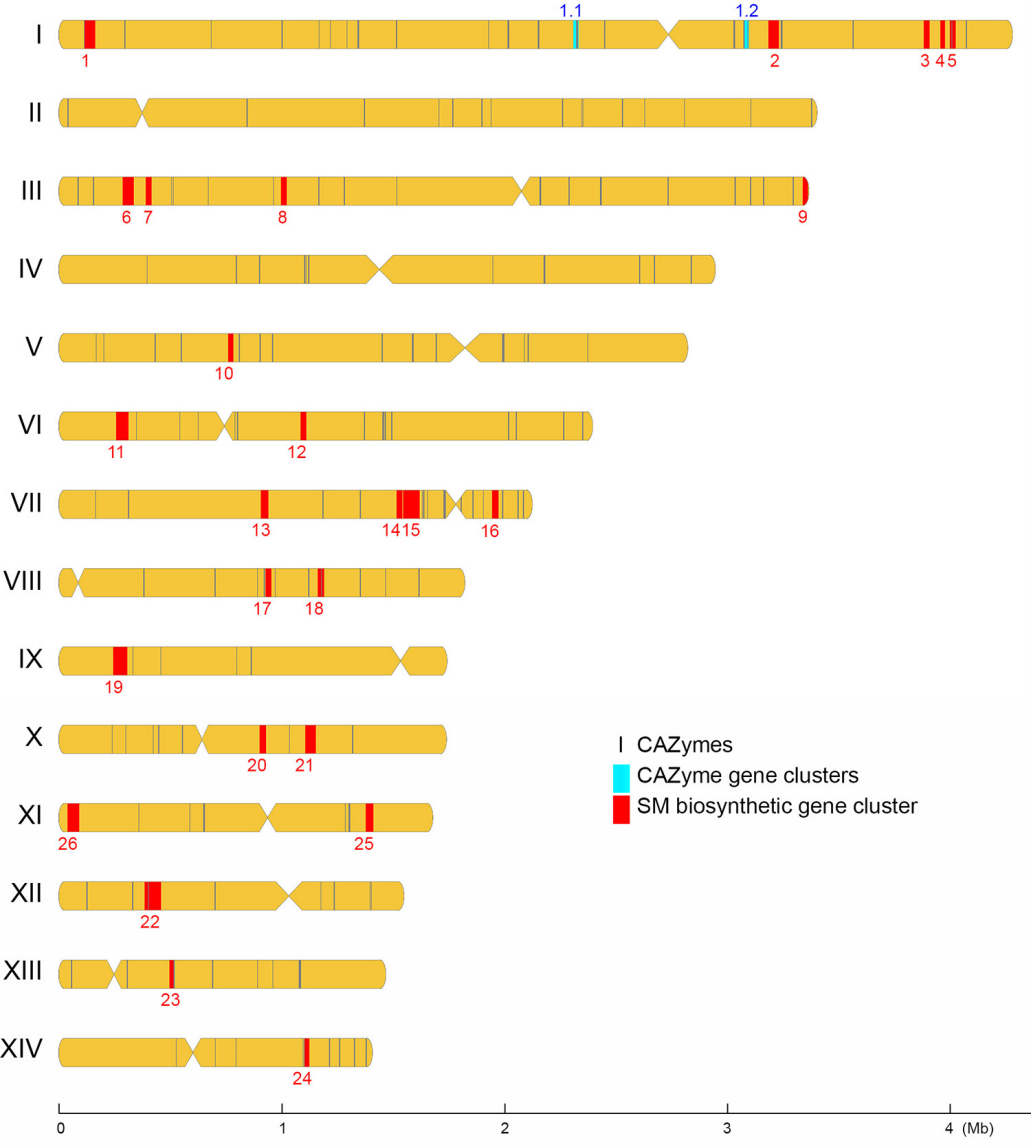
Chromosomal distribution of CAZyme genes, CAZ-GCs, and SM-BGCs in *T. camphoratus* W1. Centromere locations are shown by restricted width. All CAZyme genes were chosen along the sequence to be used as location markers. The locations of individual CAZyme genes, 2 CAZyme gene clusters (CAZ-GCs), and 26 secondary metabolite biosynthetic gene clusters (SM-BGCs) are indicated in black, cyan, and red, respectively.

There is considerable evidence showing that CAZymes cooperate with other CAZymes and signature proteins (e.g., transporters and transcriptional factors), and the respective genes tend to form physically linked CAZyme gene clusters (CAZ-GCs) in polysaccharide utilization loci (PUL) (28). To identify potential CAZ-GCs in *T. camphoratus*, we have developed high-stringency predictive software (termed ‘IMB-CAZGC”), which identified two (Fig. 6), five, three, and six CAZ-GCs in W1, W2, V5, and V7, respectively (Table S13 and Data Set 12 to 15). These results provide a comprehensive basis for further exploring CAZymes and PUL in this medicinal mushroom.

### Secondary metabolite biosynthetic genes and biosynthetic gene clusters.

Wild and basswood-cultivated *T. camphoratus* mushrooms contain high contents of natural medicinal products. It is important to identify the secondary metabolite biosynthetic genes (SM-BGs) and the biosynthetic gene clusters (SM-BGCs).

**(i) Ergosterol biosynthesis.** Ergosterol is an essential component of fungal cell membranes that determines the fluidity, permeability and activity of membrane-associated proteins (29). The *T. camphoratus* genomes not only encode all yeast Erg orthologs, but also have undergone gene multiplication or duplication. For example, there are 2 Erg1, 28 Erg5, 11 Erg6, 26 Erg11, 2 Erg20, 2 Erg24, and 4 Erg26 orthologs in the W1 genome (Table S14). Erg5 and Erg11 belong to the cytochrome P450 superfamily proteins (CYPs).

**(ii) CYPs.** The genomes of *G. lucidum* (30) and *S. commune* (31) harbor 204 and 111 CYP genes, respectively. Here, we used the widely used metagenomics web server WebMGA (32) to perform a genome-wide search for CYPs, which revealed 96, 71, 74, 75, 79, 77, 197, 114, and 149 known CYP subfamily genes in the genomes of s27 (7), s27 (by Funannotate, this study), W1, W2, V5, V7, *G. lucidum*, *S. commune* and *P. ostreatus*, respectively (Table S15). Due to errors associated with NGS-based sequencing and assembly, the original s27 genome annotation data set (7) contains spurious CYPs. Our annotation data have revealed several new CYPs in *T. camphoratus* genomes, including CYP61, CYP502, CYP613, CYP665, CYP5065, CYP5137, and CYP5155. We also performed genome-wide transcriptomic analyses to identify CYP genes that are transcriptionally upregulated (FC ≥ 3; *P < *0.05) in FBs or in monokaryotic and dikaryotic vegetative mycelia (Table S15 and Fig. S13).

**(iii) Secondary metabolite biosynthetic gene clusters.** We annotated 27 putative SM-BGCs (Data Set 16 to 19), 25 of which existed in all four *T. camphoratus* monokaryons. We identified a V5-specific SM-BGC and another SM-BGC that only exists in W1 and V7 (Table S16). The 26 SM-BGCs in W1 comprise 320 putative biosynthetic genes (BGs) (Fig. 6). Genome-wide transcriptomic analyses (FC ≥ 3 and *P* < 0.05) revealed that 37 and 63 SM-BGCs are transcriptionally upregulated in FBs and in vegetative monokaryons or dikaryons, respectively. Six SM-BGCs have ≥3 genes transcriptionally upregulated in FBs (Fig. S14).

**(iv) Differentially expressed genes in the orange-red *T. camphoratus* strains.** A polyketide synthase gene (*pks63787*) has been implicated in the biosynthesis of pigments and metabolites related to antioxidant activity because the *pks63787*Δ monokaryon not only exhibited a reduced red phenotype compared to the wild-type strain but also displayed less 1,1-biphenyl-2-picrylhydrazyl free radical scavenging activity (33). The protein product of *pks63787* shares 99% amino acid sequence identity with that of *Ac*W1_3924, a *pks* gene we identified in BGC_26. However, our data indicate that transcripts of *Ac*W1_3924 were not significantly upregulated in orange-red vegetative mycelia (V5, V7, V5×V7, and W1×V7) relative to milky-white vegetative mycelia (W1, W2, and W1×W2).

We then analyzed differently expressed genes (DEGs) (FC ≥3 and *P* < 0.05) using RNA-seq data sets from three biologically distinct samples (Fig. S15). Compared with milky-white strains (W1, W2, and W1×W2), we identified 37 upregulated genes in orange-red strains (V5, V7, and V5×V7), including an *ERG5*-like CYP gene and four dehydrogenease genes. GO enrichment indicated that these upregulated genes were mainly involved in the processes of oxidation-reduction, cellular alcohol, and aldehyde metabolism (Table S17). Using STRING (Search Tool for the Retrieval of Interacting Genes/Proteins; https://string-db.org/), we found that the S. cerevisiae orthologs (e.g., *ERG5*, *ALD5*, *AAD4*, YPL088W, and *FAS2*) of these upregulated genes were required for biosynthesis of ergosterol, lipids, and small molecules (Fig. S16). Notably, one of the upregulated genes (*Ac*W1_7136) encodes an aldehyde dehydrogenase that is highly similar in amino acid sequence to human ALDH8A1 (Aldehyde Dehydrogenase 8 Family Member A1). ALDH8A1 plays a role in the 9-cis-retinoic acid biosynthesis pathway (oxidizing 9-cis retinol to 9-cis retinoid acid) (34) or the kynurenine pathway in tryptophan catabolism (oxidizing 2-aminomuconate semialdehyde to 2-aminomuconic acid) (35). Further investigations will reveal if retinoid- or tryptophan-derived chromophores are functionally relevant to the red phenotype of orange-red *T. camphoratus* mycelia and mushrooms given that the alternating C = C double bonds in polyene side chains are responsible for the color of retinoids (typically yellow, orange, or red) and oxidation products of tryptophan also result in yellow coloration (36).

**(v) A *T. camphoratus*-specific dioxygenase gene.** We identified a dioxygenase gene (*Ac*W1_0042) peculiar to *T. camphoratus* when its genome was compared with those of several other fungal model organisms, such as *C. cinerea*, *G. lucidum* and *S. commune* (Table S18). Its closest ortholog in the filamentous fungal model organism Neurospora crassa is the carotenoid cleavage dioxygenase CAO-2. CAO-2 is a carotene torulene cleavage enzyme essential for the biosynthesis of the apocarotenoid pigment neurosporaxanthin (37). Neurosporaxanthin and its carotene precursors give N. crassa surface cultures their orange pigmentation. Biosynthesis of neurosporaxanthin in N. crassa requires three other enzymes: a bifunctional lycopene cyclase/phytoene synthase (*al-1*; NCU00585), a phytoene desaturase (al-2; NCU00552), and an aldehyde dehydrogenase (ylo-1; NCU04013) (38). *T. camphoratus* possesses three *ylo-1* gene orthologs, but lacks orthologs of *al-1* and *al-2* (Table S18). Neither *cao-2* nor *ylo-1* orthologs in *T. camphoratus* exhibit significant transcriptional differences between vegetative mycelia and FBs (Data Set 20).

**(vi) Fungal genes absent from *T. camphoratus* genomes**. *T. camphoratus* genomes lacked orthologs of the N. crassa ergot alkaloid biosynthesis protein A gene (*eabA*; NCU0747). Notably, *eabA* orthologs exist in the genomes of *C. cinerea* and *S. commune* (Table S18). The EabA protein is functionally and structurally similar to the agroclavine synthase (EasG) in *Claviceps purpura*, the festuclavine synthase (FgaFS) in Aspergillus fumigatus, and the pyroclavine synthase (FgaFS) in *Penicillium commune*. In *C. purpura*, EsaG catalyzes the conversion of chanocvalvine-I aldehyde (CIA) into agrocalvaine via a non-enzymatic adduct with reduced glutathione. In A. fumigatus and *P. commune*, FgaFS converts CIA into festuclavine and pyroclavine via FgaOx3 (CIA reductase; NCU04452). CIA is an antioxidative compound derived from L-tryptophan via sequential activity of five C. purpura enzymes, including 4-dimethylallyltryptophan synthase (DMATS; NCU12075), 4-dimethylallyltryptophan N-methyltransferase (EasF; NCU04343), FAD-dependent oxidoreductase (EasE; NCU07619), catalase (EasC; NCU05169), and short-chain dehydrogenase/reductase (EasD; NCU02128) (39). Apart from an orthologous gene to EabA/EasG/FgaFS, *T. camphoratus* genomes possess orthologs of DMATS, EasF, EasE and EasD (Table S18). Interestingly, *T. camphoratus* orthologs of both EasF and FgaOx3 are FRGs, i.e., they are transcriptionally downregulated in FBs (Data Set 20).

### LncRNAs associated with fruiting and vegetative growth.

Recent evidence indicates that lncRNAs play important roles in fungal cellular processes, including sexual development, secondary metabolism, and transcriptional regulation (40, 41). Through comparative genome-wide transcriptomic analyses (FC ≥ 3 and *P* < 0.05), we identified 188 and 278 lncRNAs (FC ≥ 3 and *P* < 0.05) that are transcriptionally upregulated in FBs (i.e., fruiting-related lncRNAs [FRLRs]) and in vegetative monokaryons or dikaryons (vegetative hyphae-related lncRNAs [VHLRs]), respectively (Fig. 5D, Fig. S17, and Data Set 21).

## DISCUSSION

In this study, we established a sexual crossing method for classical genetic studies of *T. camphoratus*, enabling us to determine and annotate high-quality and chromosome-level genomic sequences of two dikaryons, SN1 and HC1. We show that *T. camphoratus* has a tetrapolar mating-type system. Our genomic data also reveals that SN1 and HC1 represent two intraspecies isolates displaying a distinct color phenotype and genomic characteristics, including mating type idiomorphs, karyotype heterogeneity and rDNA copy number variations. RT-mediated chromosomal translocation or inversion events are responsible for karyotype heterogeneity.

Because all recognizable vegetative colonies derived from wild and artificially cultivated *T. camphoratus* mushrooms were mostly likely germinated from arthrospores rather than basidiospores, further investigations will be needed to reveal optimal conditions or substrates for basidiospore germination. An alternative possibility is that most (if not all) of the basidiospores we collected from wild and artificially cultivated *T. camphoratus* mushrooms were inviable. That hypothesis raises an interesting conjecture that, due to extensive expansion of RTs, *T. camphoratus* genomes might have lost the capability to carry out proper pairing, recombination or synapsis between homologous non-sister chromosomes during meiotic prophase. Failure of maternal and paternal chromosomes to pair, recombine, and connect is arguably the most important cause of meiotic nondisjunction, which results in sperm, oocytes, or fungal sexual spores having abnormal chromosome numbers (aneuploidy) (42). Such meiotic abnormalities might result in decreased levels of basidiospore viability.

Compared with the genomes of other fungal model organisms (e.g., *C. cinerea*, *G. lucidum*, *S. commune*, and *P. ostreatus*), the genomes of *T. camphoratus* mushrooms have lost a panel of enzymes that degrade plant, fungal, and bacterial cell walls. This finding provides useful clues explaining several unique features of *T. camphoratus* mushrooms: (i) it is difficult and time-consuming to artificially cultivate *T. camphoratus* mushrooms on *Ck* basswood; (ii) wild *T. camphoratus* mushrooms are rare and often fruit on rotten *Ck* tree hearts; and (iii) bacterial and fungal infections frequently occurred on *T. camphoratus* vegetative cultures or during mushroom cultivation. Our comparative genomic analyses have also revealed two key findings related to SM-BGs or SM-BGCs in *T. camphoratus*. First, the four nearly complete *T. camphoratus* genome sequences possess a collection of CYP genes that were either not or mistakenly annotated in the original s27 genome (7). Second, the *T. camphoratus* genomes exhibit a significant contraction in numbers of SM-BG families and individual genes. This latter result is somewhat surprising because *T. camphoratus* mushrooms are known to harbor 10 times more natural products (i.e., benzenoids, benzoquinones, and triterpenoids) than *G. lucidum* (2). It remains unclear if this unique property of SM-BG deficit in *T. camphoratus* is associated with or even contributes to the pronounced accumulation of natural products in *T. camphoratus* mushrooms. It is also possible that some natural products found in wild and basswood-cultivated *T. camphoratus* mushrooms are xenobiotics or metabolic by-products of xenobiotics. Xenobiotics are chemicals found within an organism but not naturally produced by it. Taken together, the most reasonable inference from our comparative genomic analyses is that *T. camphoratus* might have undergone retrogressive evolution, which could (at least partly) explain many of the distinctive properties of this economically important mushroom.

*T. camphoratus* is a native Taiwanese medicinal mushroom which has been widely used as a complementary and alternative medicine in many other countries. The classical breeding method and comprehensive genomic data sets we report herein are valuable resources for new innovations in mushroom cultivation and biosynthesis of natural medicinal products. Our results of comparative genomic analyses not only have significantly extended our knowledge of this economically important basidiomycete, but also for mushrooms in general.

## MATERIALS AND METHODS

### Fungal strains.

All four monokaryons have been submitted to the Bioresource Collection and Research Center at the Food Industry Research and Development Institute, Hsin-Chu, Taiwan (Republic of China), which is accessible at https://www.bcrc.firdi.org.tw/en/home/. The strain ID numbers are BCRC-MU30395 (V5), BCRC-MU30396 (V7), BCRC-MU30397 (W1), and BCRC-MU30398 (W2). All other strains (e.g., V2, Q7, W1×W2, V5×V7, and W1×V7) are available from the corresponding authors upon reasonable request.

### Miscellaneous.

The vegetative mycelia of all *T. camphoratus* monokaryons and dikaryons were grown on Malt Extract Agar at 25°C and stored at −80°C in 30% glycerol. The mycelia of the SN1 and HC1 dikaryons undergo vegetative growth via clamp connections. In contrast, all six monokaryons do not possess clamp connections. The arthrospores were collected by using a sterile loop after culturing for 5 to 7 days. Cultivation of *T. camphoratus* mushrooms on aged *Ck* basswood was carried out by Chung-Yu Chen at the Shen Nong Fungal Biotechnology Co. Ltd. (Taoyuan, Taiwan). Some basswood-cultivated *T. camphoratus* mushrooms used in this study were provided by KFK Biotech Co. Ltd. (Kaohsiung, Taiwan) and the HIMA Foundation (Taipei, Taiwan), a non-governmental and non-profit making society for environmental protection. Preparation of high-quality genomic DNA and RNA, PCR genotyping, Southern hybridization, DAPI (4′,6-diamidino-2-phenylindole) staining, and cytological analysis were carried out as described previously (8, 9, 43). The *Ck* basswood used for mushroom cultivation was moistened with an ∼50% volume of ddH_2_O that had been autoclaved and then cooled to 25°C. Next, the *Ck* basswood was planted with vegetative mycelia and sealed in a breathable plastic bag. Cultivation was carried out in a 25°C dark room with 70% to 80% humidity for 3 to 6 months. The basswood was then removed from the plastic bag and subjected to continuous incubation under the same conditions until FBs emerged from the basswood. The basidia and basidiospores of *T. camphoratus* mushrooms observed by scanning electron microscopy (FEI Quanta 200) with a cryogenic system (Quorum PP2000TR FEI) at the Cell Biology Core Laboratory of the Institute of Plant and Microbial Biology, Academia Sinica.

### High-throughput DNA/RNA sequencing, genome assembly, annotation, and evaluation.

Whole genome sequencing and assembly were carried out using the PacBio SMRT method and the Illumina-MiSeq paired-end method (8, 9, 43) (Table S2 to S5). The Illumina-MiSeq paired-end method was also applied to generate high-quality RNA-seq data sets of four monokaryotic vegetative mycelia (V5, V7, W1, W2), two dikaryotic vegetative mycelia (V5×V7 and W1×W2), and three basswood-cultivated FBs (V5×V7, W1×V7, and W1×W2), respectively (Fig. 1). All RNA-seq data sets listed in Table S7 were included for genome-wide prediction of protein-coding genes using the Funannotate v1.8.1 pipeline, as described previously (9). The “Trinity” software tool was applied for *de novo* transcriptome assembly (44). BUSCO (https://busco.ezlab.org), an open-source software with a large selection of lineage-specific sets of Benchmarking Universal Single-Copy Orthologs, was applied to quantitatively measure genome assembly and annotation completeness (45). Selected evolutionarily-informed expectations of gene content from near-universal single-copy orthologs were obtained from OrthoDB (https://www.orthodb.org) (46).

Identification and annotation of long non-coding RNAs (lnRNAs) were performed as described previously (47). In brief, NGS-based RNA reads were trimmed and mapped to the nearly complete *T. camphoratus* genome sequences using HISAT2 (hierarchical indexing for spliced alignment of transcripts) with default setting (http://daehwankimlab.github.io/hisat2/) (48). The transcripts were then assembled using StringTie software (49) and using the available *T. camphoratus* annotation as the reference (this study). After reconstruction analysis of the transcripts, Cuffcompare (50) was used to compare the corresponding transcripts to known gene models, and then corresponding information on positional relationships was obtained. The initial transcripts were compared with known genes using Cuffcompare, and only transcripts at non-gene loci were selected for further analysis. Next, the assembled transcripts with length < 200 and FPKM ≦ 1 were excluded. The assembled transcripts with coding potential were removed according to the evaluations of three software tools: Coding Potential Calculator, txCdsPredict, and Coding-Non-Coding Index. The assembled transcripts, including known protein domains, were also removed, following their comparison with sequences in the Pfam protein family database. Finally, the transcripts were predicted by at least three prediction tools were considered high-confidence lncRNA candidates (51–53).

### Pulsed-field gel electrophoresis.

Intact chromosomal DNA was prepared using the agarose spheroplasting method with two modifications. The arthrospores were collected from the Malt Extract Agar plates at 25°C, and embedded into the agarose plug. *Trichoderma harziaum* lysing enzymes (Sigma-Aldrich Co., St. Louis, MO) were used to digest the cell walls during spheroplasting. Electrophoresis conditions for karyotype analysis were described previously (8, 9).

### Repetitive sequence annotation.

Novel repeat elements were first identified in RepeatModeler-1.0.4 (http://www.repeatmasker.org/RepeatModeler.html) with default parameters. RepeatMasker (version 4.0.6) and the Repbase Library (http://www.repeatmasker.org) were used to scan the four different *T. camphoratus* haploid genomes for interspersed repeats and low-complexity DNA sequences (54). To obtain high-confidence data, the preliminary RepeatMasker data were then filtered with two parameters (length ≥140, Smith–Waterman local similarity scores ≥450) (8, 9). Using these two parameters, we were able to identify almost all transposable elements along the 16 chromosomes of S. cerevisiae (8). To identify LTR-RTs in the *T. camphoratus* centromeric regions, we also applied the LTR-finder program (http://tlife.fudan.edu.cn/tlife/ltr_finder/) (55).

### Comparative genomics and protein family analyses.

For comparative genomic analyses, the protein-coding gene models of four representative fungi were downloaded from the corresponding databases: Coprinopsis cinerea (http://fungi.ensembl.org/Coprinopsis_cinerea_okayama7_130_gca_000182895/Info/Index?db=core;g=CC1G_13912;r=2:1555966-1559452;t=EFI28378), Ganoderma lucidum (https://mycocosm.jgi.doe.gov/Gansp1/Gansp1.home.html), *Schizophyllum commune* (https://mycocosm.jgi.doe.gov/Schco1/Schco1.home.html), and Pleurotus ostreatus (https://mycocosm.jgi.doe.gov/PleosPC15_2/PleosPC15_2.home.html), respectively.

Genome-wide prediction of genes and gene families was carried out using the Funannotate v1.8.1 pipeline (56). Carbohydrate-active enzymes (CAZymes) were reannotated using the dbCAN2 meta server (http://bcb.unl.edu/dbCAN2) (21). We have developed the software tool “IMB-CAZGC” to predict potential CAZyme gene clusters (CAZ-GCs) with the following assumptions. Specifically, a potential CAZ-GC must contain ≥3 CAZyme genes or ≥2 CAZyme genes with ≥1 other specific signature genes, namely, transporters or transcriptional factors. Also, the CAZyme genes or the other signature gene(s) must be within two intergenic distances of the CAZyme genes. Although the prediction requirements in IMB-CAZGC are more stringent than those in dbCAN2 (21) and dbCAN-PUL (57), IMB-CAZGC was able to identify all four previously identified CAZ-GCs in Trichoderma reesei QM6a, the ancestor of all currently used cellulase-producing mutants (58). WebMGA (http://weizhong-lab.ucsd.edu/webMGA/), a customizable web server for fast metagenomic sequence analysis (32), was used to identify cytochrome P450 (CYP) subfamilies in the Eukaryotic Orthologous Groups (KOG) database by reverse PSI-BLAST (RPS-BLAST) (59). The results revealed five different NCBI KOG ID groups: KOG0156 (CYP2 subfamily), KOG0157 (CYP4/CYP19/CYP26 subfamilies), KOG0158 (CYP3/CYP5/CYP6/CYP9 subfamilies), KOG0159 (CYP11/CYP12/CYP24/CYP27 subfamilies), and KOG0684 (a unique gene). Finally, all putative CYP genes were scanned through the Fungal Cytochrome P450 Database (p450.riceblast.snu.ac.kr/index.php?a = view) to reveal each of their CYP subfamilies, respectively (60). Funannotate (56) not only parses protein-coding models from the annotation, but also identifies numbers and classes of CAZymes, proteases, transcriptional factors, secreted proteins, polyketide synthases (PKSs), and nonribosomal peptide synthetases (NRPSs). To predict secreted proteins, we downloaded the SignalP 4.1 (61), TMHMM 2.0 (62), and big-PI Fungal Predictor (63) programs into “Funannotate” and then applied them with default settings. In this study, effector candidate proteins were defined as predicted secreted proteins (with a signal peptide present, but no transmembrane domains or glycosylphosphatidylinositol anchors) having lengths of <300 amino acids. To assess if effector candidates presented similarity to known proteins, we performed BLASTP analysis (cutoff E-value 10^−5^) using the Swiss-Prot database (downloaded October 22, 2016).

To identify evolutionarily conserved FRGs in the four *T. camphoratus* haploid genomes, we applied standard protein-protein BLAST (e-value ≤10^−^**^17^**; [21]) using genes that had previously been shown to play a role in mushroom formation of several other fungi, including *Agrocybe aegerita*, *C. cinerea*, and *S. commune* (20, 64–81).

### RNA-seq, bioinformatics, and comparative transcriptomic analyses.

Total RNA was extracted from cultured vegetative mycelia and basswood-cultivated mushrooms using TRIzol (Invitrogen) as described recently (9). RNA quality and quantifications were determined using an Agilent 2100 Bioanalyzer. The mRNA sequencing libraries were prepared using a Truseq Stranded mRNA kit (Illumina), and 75 to 76 cycle single-read sequencing was performed using the 500 High-output v2 (75 cycle) sequencing kit on an Illumina NextSeq500 instrument. Adaptor sequences were removed from raw sequences and then quality was examined by FastQC software. Bioinformatic analysis was performed as described previously (8, 9). HISAT2 was applied for sequence mapping (GRCm38.p6) with default settings (48), featureCounts for counting reads (82), and classic EdgeR and Fisher’s exact test for differential expression and statistical analysis (83). To analyze results from both systems, we first set a false discovery rate (FDR) of *P* < 0.05 to select significantly altered gene expression. Next, we stipulated a fold change (FC) of ≥3 or ≤3 for up- and downregulated genes, respectively. To remove genes with background expression levels, the average transcripts per kilobase million (TPM) in one of the groups had to be > 0.5. TPM = (individual gene RPK/the sum of all RPKs) × 10^6^, whereas reads per kilobase (RPK) = (read counts/transcription length). The raw data have been deposited online. The Trinity platform (http://trinityrnaseq.sourceforge.net.) was used to extract clusters of transcripts with similar expression profiles by cutting the transcript cluster dendrogram at 30% of its height (84).

### Data availability.

PacBio raw reads, RNA-seq paired-end data sets, the final genome assembly results, and the gene annotation results have been submitted to the National Center for Biotechnology Information (NCBI) under the BioProject PRJNA386064 and BioProject PRJNA615295 (https://www.ncbi.nlm.nih.gov/bioproject/), respectively. The supplemental information file includes 18 supplemental tables and 17 supplemental figures. Twenty-one supplemental data sets (Data Set 1 to 21) and the source code of the “IMB-CAZGC” software tool are also publicly available at https://github.com/tfwangasimb/tfwangasimb-Supplemental-data-and-dataset-for-the-near-complete-genomes-sequences-of-Antrodia-cinna/releases.
