# Supplementary material for: Sexual Crossing, Chromosome-Level Genome Sequences, and Comparative Genomic Analyses for the Medicinal Mushroom Taiwanofungus Camphoratus (Syn. Antrodia Cinnamomea, Antrodia Camphorata)
Source: Microbiol Spectr. 2022 Feb 23;10(1):e02032-21. doi: 10.1128/spectrum.02032-21 (PMC8865532; doi:10.1128/spectrum.02032-21)
Supplement: SUPPLEMENTAL FILE 1 — Supplemental material. Download SPECTRUM02032-21_Supp_1_seq10.pdf, PDF file, 2.4 MB [file spectrum02032-21_supp_1_seq10.pdf]

Sexual crossing, chromosome-level genome sequences, and comparative genomic analyses for the medicinal mushroom *Taiwanofungus camphoratus* (syn. *Antrodia cinnamomea*, *Antrodia camphorata)*

Chia-Ling Chen[1], Wan-Cheng Li[1], Yu-Chien Chuang[1], Hou-Chen Liu[1], Chien-Hao Huang[1], Ko-Yun Lo[1], Chung-Yu Chen[2], Fang-Mo Chang[3], Guo-An Chang[4], Yu-Ling Lin[5], Wen-Der Yang[4], Ching-Hua Su[6], Tsung-Ming Yeh[2], and Ting-Fang Wang[1,#]

[1]Institute of Molecular Biology, Academia Sinica, Taipei 115, Taiwan
[2]Shen Nong Fungal Biotechnology Co. Ltd., 50-2, Zhongpu 6th St., Taoyuan Dist., Taoyuan City 330, Taiwan
[3]School of Dentistry, College of Oral Medicine, Taipei Medical University, Taipei 110, Taiwan
[4]KFK Biotech Co. Ltd., 29 Haibian Rd., Ling-Ya Dist., Kaohsiung 802, Taiwan
[5]HIMA Foundation, 5-4 Lane 12, Alley 190, Sc. 7, ChungShen N. Rd., Shilin Dist. Taipei 111, Taiwan
[6]Department of Microbiology and Immunology, Taipei Medical University, Taipei 110, Taiwan
Correspondence to Ting-Fang Wang (email: tfwang@gate.sinica.edu.tw)

The supplemental information (*SI*) file that includes 18 supplemental tables and 17 supplemental figures.

Twenty one supplemental datasets (*SD*, DS1-DS21) and the source code of the 'IMB-CAZGC' software tool are publicly available at https://github.com/tfwangasimb/Supplemental-data-and-dataset-for-the-near-complete-genomes-sequences-of-Antrodia-cinnamomea/releases/download/20211025Ac/SI and DS.zip.

Table S1. List of all *T. camphoratus* monokaryons analyzed by PacBio RSII and Illumina-Nextseq whole genome sequencing technology

Strain	Ploidy	Sequencing platform	BioProject accession number	Source
W1	Monokaryon	PacBio RSII	PRJNA386064	CLC
W2	Monokaryon	PacBio RSII	PRJNA386064	CLC
V5	Monokaryon	PacBio RSII	PRJNA386064	CHS
V7	Monokaryon	PacBio RSII	PRJNA386064	CHS
W1	Monokaryon	Illumina Nextseq 500	PRJNA615295	CLC
W2	Monokaryon	Illumina Nextseq 500	PRJNA615295	CLC
V5	Monokaryon	Illumina Nextseq 500	PRJNA615295	CHS
V7	Monokaryon	Illumina Nextseq 500	PRJNA615295	CHS

Table S2. Chromosomes or scaffolds terminating in typical telomeric sequences for the *T. camphoratus* W1 monokaryon

Chromosome	Funannotate	Pac-Bio Assembly	Length (bps)	Telomere		Remarks
				5’	3’	
ChI	scaffold_1	unitig_0	4278167	+	+	
ChII	scaffold_2	unitig_2	3401759	+	+	
ChIII	scaffold_3	unitig_1	3365326	+	+	
ChV	scaffold_4	unitig_6	2821609	+	+	
ChVI	scaffold_5	unitig_8	2394950	+	+	
ChVII	scaffold_6	unitig_65	2123879	+	-	rDNA repeat
ChIVA	scaffold_7	unitig_84	2082428	+	-	
ChVIII	scaffold_8	unitig_10	1821498	+	+	
ChIX	scaffold_9	unitig_11	1741554	+	+	
ChX	scaffold_10	unitig_12	1738909	+	+	
ChXII	scaffold_11	unitig_15	1548230	+	+	
ChXIII	scaffold_12	unitig_17	1466623	+	+	
ChXIV	scaffold_13	unitig_18	1406655	+	+	
ChXIB	scaffold_14	unitig_85	1226853	-	+	
ChIVB	scaffold_15	unitig_5	878346	-	+	
ChXIA	scaffold_16	unitig_14	474026	-	+	
Contig1	scaffold_17	unitig_69	48768	-	-	rDNA repeat
Contig2	scaffold_18	unitig_7	42349	-	-	
Contig3	scaffold_19	unitig_75	37703	-	-	rDNA repeat
Contig4	scaffold_20	unitig_20	35276	-	-	
Contig5	scaffold_21	unitig_72	35244	-	-	rDNA repeat
Contig6	scaffold_22	unitig_71	34410	-	-	rDNA repeat
Contig7	scaffold_23	unitig_76	34195	-	-	rDNA repeat
Contig8	scaffold_24	unitig_66	34100	-	-	rDNA repeat
Contig9	scaffold_25	unitig_78	33596	-	-	rDNA repeat
Contig10	scaffold_26	unitig_3	32035	-	-	
Contig11	scaffold_27	unitig_68	30952	-	-	rDNA repeat
Contig12	scaffold_28	unitig_16	30724	-	-	
Contig13	scaffold_29	unitig_22	30210	-	-	rDNA repeat
Contig14	scaffold_30	unitig_73	28884	-	-	rDNA repeat
Contig15	scaffold_31	unitig_79	28211	-	-	rDNA repeat
Contig16	scaffold_32	unitig_23	24180	-	+	rDNA repeat
Contig17	scaffold_33	unitig_13	23500	-	-	

Contig18	scaffold_34	unitig_80	20475	-	-	rDNA repeat
Contig19	scaffold_35	unitig_82	17485	-	+	rDNA repeat

Table S3. Chromosomes or scaffolds terminating in typical telomeric sequences for the *T. camphoratus* W2 monokaryon

Chromosome	Funannotate	PacBio Assembly	Length (bps)	Telomere		Remark
				5'	3'	
ChI	scaffold_1	unitig_0	4123433	+	+	
ChIII	scaffold_2	unitig_1	3268603	+	+	
ChV	scaffold_3	unitig_6	3052342	+	+	
ChIIA	scaffold_4	unitig_76	3035724	-	+	
ChVI	scaffold_5	unitig_7	2450889	+	+	
ChIVB	scaffold_6	unitig_74	2390429	+	-	
ChVII	scaffold_7	unitig_77	2125258	+	-	rDNA repeat
ChVIII	scaffold_8	unitig_9	1874230	+	+	
ChX	scaffold_9	unitig_14	1679038	+	+	
ChXII	scaffold_10	unitig_15	1655562	+	+	
ChIXB	scaffold_11	unitig_2	1629413	+	-	
ChXIII	scaffold_12	unitig_16	1499514	+	+	
ChXIV	scaffold_13	unitig_17	1421868	+	+	
ChXIA	scaffold_14	unitig_11	1013923	-	+	
ChXIB	scaffold_15	unitig_13	709686	+	-	
ChIVA	scaffold_16	unitig_4	625946	-	+	
ChIIB	scaffold_17	unitig_3	281240	-	+	
Contig1	scaffold_18	unitig_12	126676	-	-	
ChIXA	scaffold_19	unitig_20	92376	+	-	
Contig2	scaffold_20	unitig_80	59265	-	-	rDNA repeat
Contig3	scaffold_21	unitig_8	58983	-	-	
Contig4	scaffold_22	unitig_10	50297	-	-	
Contig5	scaffold_23	unitig_75	45112	-	-	
Contig6	scaffold_24	unitig_72	42627	-	-	rDNA repeat
Contig8	scaffold_25	unitig_23	36387	-	-	rDNA repeat
Contig9	scaffold_26	unitig_73	35555	-	-	rDNA repeat
Contig10	scaffold_27	unitig_78	35085	-	-	rDNA repeat
Contig11	scaffold_28	unitig_81	34950	-	-	rDNA repeat
Contig12	scaffold_29	unitig_71	33715	-	-	rDNA repeat
Contig13	scaffold_30	unitig_70	30570	-	-	rDNA repeat
Contig14	scaffold_31	unitig_22	29451	+	-	rDNA repeat
Contig15	scaffold_32	unitig_21	23760	-	-	

Table S4. Chromosomes or scaffolds terminating in typical telomeric sequences for the *T. camphoratus* V5 monokaryon

Chromosome	Funannotate	PacBio Assembly	Length (bps)	Telomere		Remark
				5’	3’	
ChII	scaffold_1	unitig_0	4545385	+	+	
ChI	scaffold_2	unitig_4	4069135	+	+	
ChIII	scaffold_3	unitig_7	3292975	+	+	
ChIV	scaffold_4	unitig_3	2482552	+	-	
ChVI	scaffold_5	unitig_10	2407266	+	+	
ChVII	scaffold_6	unitig_37	2087226	+	-	rDNA repeat
ChVA	scaffold_7	unitig_6	1811452	+	-	
ChXIII	scaffold_8	unitig_16	1593142	+	+	
ChXII	scaffold_9	unitig_19	1580306	+	+	
ChXIV	scaffold_10	unitig_17	1466462	+	+	
ChXI	scaffold_11	unitig_32	1222392	+	-	
ChVIIIB	scaffold_12	unitig_34	1193591	+	-	
ChVB	scaffold_13	unitig_8	977763	-	-	
ChIXB	scaffold_14	unitig_36	807467	-	+	
ChIXA	scaffold_15	unitig_18	780258	-	+	
ChVIIIA	scaffold_16	unitig_15	649039	+	-	
Contig1	scaffold_17	unitig_9	532859	+	-	
ChX	scaffold_18	unitig_24	488068	+	+	
Contig2	scaffold_19	unitig_33	289346	-	+	
ChVC	scaffold_20	unitig_21	224197	+	-	
ChII	scaffold_21	unitig_38	90961	-	-	rDNA repeat
Contig4	scaffold_22	unitig_13	41333	-	-	rDNA repeat
Contig5	scaffold_23	unitig_39	35356	-	-	rDNA repeat
Contig6	scaffold_24	unitig_2	34427	-	-	
Contig7	scaffold_25	unitig_14	33381	-	-	rDNA repeat
Contig8	scaffold_26	unitig_5	23324	-	-	
Contig9	scaffold_27	unitig_20	22606	-	-	
Contig10	scaffold_28	unitig_22	21583	-	-	
Contig11	scaffold_29	unitig_11	18501	-	-	
Contig12	scaffold_30	unitig_1	18065	-	-	
Contig13	scaffold_31	unitig_35	8060	-	-	
Contig14	scaffold_32	unitig_26	7118	-	-	

Table S5. Chromosomes or scaffolds terminating in typical telomeric sequences for the *T. camphoratus* V7 monokaryon

Chromosome	Funannotate	Pac-Bio Assembly	Length (bps)	Telomere		Remark
				5'	3'	
ChIB	scaffold_1	unitig_0	4147377	+	-	
ChIVA	scaffold_2	unitig_4	2105587	+	-	
ChVII	scaffold_3	unitig_88	2020100	+	-	rDNA repeat
ChVB	scaffold_4	unitig_6	1967586	-	+	
ChIIA	scaffold_5	unitig_1	1929505	+	-	
ChIIIC	scaffold_6	unitig_2	1772042	-	-	
ChIIIA	scaffold_7	unitig_9	1368267	-	-	
ChVIC	scaffold_8	unitig_75	1361003	-	-	
ChXIIA	scaffold_9	unitig_92	1247881	+	-	
ChXV	scaffold_10	unitig_12	1060187	-	+	
ChXIB	scaffold_11	unitig_11	1042070	-	-	
ChXIIIB	scaffold_12	unitig_15	1040464	-	+	
ChVIIIC	scaffold_13	unitig_8	963225	+	-	
ChIIC	scaffold_14	unitig_25	818744	+	-	
ChVA	scaffold_15	unitig_22	773809	-	+	
ChIIB	scaffold_16	unitig_5	750844	+	-	
ChXA	scaffold_17	unitig_69	702657	-	+	
ChIVB	scaffold_18	unitig_93	660001	-	+	
ChVIIIA	scaffold_19	unitig_65	640861	-	-	
ChIIIB	scaffold_20	unitig_68	627918	-	-	
ChVIA	scaffold_21	unitig_35	623544	-	+	
ChVD	scaffold_22	unitig_95	599274	+	-	
ChIXB	scaffold_23	unitig_81	598425	-	-	
ChXIIIA	scaffold_24	unitig_7	573901	-	-	
ChXIVA	scaffold_25	unitig_21	567865	-	+	
ChVID	scaffold_26	unitig_30	473097	-	+	
ChXIVB	scaffold_27	unitig_19	455160	-	+	
ChVIB	scaffold_28	unitig_17	343072	-	-	
ChXIA	scaffold_29	unitig_80	310900	-	-	
ChVIIIB	scaffold_30	unitig_14	272112	-	-	
ChIXA	scaffold_31	unitig_29	271870	-	-	
ChIA	scaffold_32	unitig_24	221158	+	-	
Contig1	scaffold_33	unitig_23	151490	-	-	

Contig2	scaffold_34	unitig_28	109275	-	-	
Contig3	scaffold_35	unitig_34	63398	-	+	
Contig4	scaffold_36	unitig_27	40319	-	-	
Contig5	scaffold_37	unitig_67	35709	-	-	
Contig6	scaffold_38	unitig_100	34427	-	-	
Contig7	scaffold_39	unitig_90	30969	-	-	rDNA repeat
Contig8	scaffold_40	unitig_84	30025	-	-	
Contig19	scaffold_41	unitig_89	29719	-	-	rDNA repeat
Contig10	scaffold_42	unitig_38	27090	-	-	rDNA repeat
Contig11	scaffold_43	unitig_16	25836	-	-	
Contig12	scaffold_44	unitig_13	23973	-	-	
Contig13	scaffold_45	unitig_33	23798	-	-	mitochondrion
Contig14	scaffold_46	unitig_86	23474	-	-	rDNA repeat
ChVC	scaffold_47	unitig_40	23259	-	-	rDNA repeat
Contig15	scaffold_48	unitig_72	22433	-	-	mitochondrion
Contig16	scaffold_49	unitig_70	21090	-	-	mitochondrion
Contig17	scaffold_50	unitig_37	20459	-	-	rDNA repeat
Contig18	scaffold_51	unitig_74	20198	-	-	
Contig19	scaffold_52	unitig_26	19635	-	-	
Contig20	scaffold_53	unitig_85	19506	-	-	
ChXIIB	scaffold_54	unitig_18	19465	-	-	
Contig21	scaffold_55	unitig_10	19272	-	-	
Contig22	scaffold_56	unitig_76	18764	-	-	mitochondrion
Contig23	scaffold_57	unitig_77	18745	-	-	mitochondrion
Contig24	scaffold_58	unitig_39	18685	-	-	
Contig25	scaffold_59	unitig_91	17866	-	-	rDNA repeat
Contig26	scaffold_60	unitig_87	17629	-	-	rDNA repeat
Contig27	scaffold_61	unitig_20	15458	-	-	
Contig28	scaffold_62	unitig_41	9784	-	-	
Contig29	scaffold_63	unitig_73	8044	-	-	mitochondrion
Contig30	scaffold_64	unitig_103	7567	-	-	
Contig31	scaffold_65	unitig_104	3504	-	-	
Contig32	scaffold_66	unitig_79	3088	-	-	mitochondrion
Contig33	scaffold_67	unitig_57	1870	-	-	Contaminate with DNA

Table S6. Summary of sequence assembly and annotation results of *T. camphoratus* genomes

<table>
<tr><th colspan="2">Strain</th><th>W1</th><th>W2</th><th>V5</th><th>V7</th><th colspan="2">s27</th></tr>
<tr><td colspan="2">Sequencing method</td><td>PacBio RSII</td><td>PacBio RSII</td><td>PacBio RSII</td><td>PacBio RSII</td><td colspan="2">Roche 454
lumina Hiseq</td></tr>
<tr><td colspan="2">Total sequenced bases (bps)</td><td>4,399,477,703</td><td>5,075,765,386</td><td>5,002,470,320</td><td>7,383,980,134</td><td colspan="2">-</td></tr>
<tr><td colspan="2">Number reads</td><td>264,784</td><td>317,227</td><td>317,159</td><td>532,329</td><td colspan="2">-</td></tr>
<tr><td colspan="2">N50 reads (bp)</td><td>22,707</td><td>21,238</td><td>21,984</td><td>19,149</td><td colspan="2">-</td></tr>
<tr><td colspan="2">Max of all assembled contigs (bp)</td><td>4,278,167</td><td>4,123,432</td><td>4,545,385</td><td>4,147,375</td><td colspan="2">2,218,769</td></tr>
<tr><td colspan="2">N50 of all assembled contigs (bp)</td><td>2,123,879</td><td>2,390,429</td><td>2,407,268</td><td>1,247,878</td><td colspan="2">1,034,879</td></tr>
<tr><td colspan="2">Contigs</td><td>36</td><td>33</td><td>33</td><td>68</td><td colspan="2">360</td></tr>
<tr><td colspan="2">Contigs (with rDNA repeats)</td><td>14</td><td>9</td><td>4</td><td>8</td><td colspan="2">-</td></tr>
<tr><td colspan="2">Coverage</td><td>120.9x</td><td>132.5x</td><td>114.1x</td><td>139.9x</td><td colspan="2">878x</td></tr>
<tr><td colspan="2">Phred Quality Score (Q).</td><td>48.7</td><td>48.5</td><td>48.7</td><td>48.7</td><td colspan="2">-</td></tr>
<tr><td colspan="2">Genome size (bp)</td><td>33,373,109</td><td>33,571,907</td><td>32,855,596</td><td>33,286,329</td><td colspan="2">32,155,604</td></tr>
<tr><td colspan="2">Mitochondrial genome (circular; bp)</td><td>115,207</td><td>115,206</td><td>107,517</td><td>107,518</td><td colspan="2">114,890</td></tr>
<tr><td colspan="2">Unidentified base (N; bp)</td><td>0</td><td>0</td><td>0</td><td>0</td><td colspan="2">-</td></tr>
<tr><td colspan="2">GC content</td><td>50.68%</td><td>50.68%</td><td>50.66%</td><td>50.67%</td><td colspan="2">50.60 %</td></tr>
<tr><td colspan="2">Long non-coding RNAs (lnRNAs)</td><td>3835</td><td>3808</td><td>3530</td><td>4007</td><td colspan="2">-</td></tr>
<tr><td rowspan="2">BUSCO genome metrics</td><td>Total</td><td>96.1 %</td><td>95.8 %</td><td>95.9 %</td><td>96.2 %</td><td colspan="2">95.4%</td></tr>
<tr><td>Single complete (S)
Duplicated complete (D)
Fragment (F)
Missing (M)</td><td>S: 96.0 %
D: 0.1 %
F: 0.8 %
M: 3.1 %</td><td>S: 95.4 %
D: 0.4 %
F: 0.8 %
M: 3.4 %</td><td>S: 95.5 %
D: 0.4 %
F: 0.9 %
M: 3.2 %</td><td>S: 95.9 %
D: 0.3 %
F: 0.7 %
M: 3.1 %</td><td colspan="2">S:95.0%
D:0.4%
F:3.4%
M:1.2%</td></tr>
<tr><td rowspan="2">BUSCO protein metrics</td><td rowspan="2">Total (%)</td><td rowspan="2">94.6 %</td><td rowspan="2">96.6 %</td><td rowspan="2">95.9 %</td><td rowspan="2">96.2 %</td><td>(1)</td><td>New</td></tr>
<tr><td>90.9 %</td><td>96.5 %</td></tr>
<tr><td colspan="2">Protein encoding genes
(Uui-genes)</td><td>10247</td><td>10273</td><td>10401</td><td>10308</td><td>9254</td><td>11809</td></tr>
</table>

Transposable elements						
Overall		1509	1465	1374	1498	Not determined
Class I RT	Tad1-LINE	2	2	2	4	
	RI-LINE	2	2	0	0	
	Jockey-LINE	1	1	0	0	
	Penelope-LINE	93	68	41	58	
	other LINEs	0	1	0	0	
	Copia-LTR	79	86	81	82	
	Gypsy-LTR	1242	1212	1157	1259	
	other LTRs	9	9	8	13	
Class II RT	CMC-EnSpm	2	2	2	2	
	MULE-MuDR	1	1	1	1	
	TcMar	3	7	8	7	
	TcMar -Sagan	2	2	2	2	
	TcMar -Tcl	2	1	1	3	
	Others	71	71	71	67	

Table S7. List of all *T. camphoratus* strains analyzed by NGS-based RNA-seq using the Illumina-Nextseq 500 sequencing platform

Strain	Sample types	BioProject accession #	Source
W1	Monokaryon	PRJNA615295	CLC
W2	Monokaryon	PRJNA615295	CLC
V5	Monokaryon	PRJNA615295	CHS
V7	Monokaryon	PRJNA615295	CHS
V2	Monokaryon	PRJNA615295	CHS
Q3	Monokaryon	PRJNA615295	CHS
V5xV7	Dikaryon	PRJNA615295	CLC
V2xQ3	Dikaryon	PRJNA615295	CLC
W1xW2	Dikaryon	PRJNA615295	CLC
V5xV7	Fruiting body	PRJNA615295	CYC
SN1	Fruiting body	PRJNA615295	CYC
W1xV7	Fruiting body	PRJNA615295	CYC
S5	Fruiting body	PRJNA615295	GAC, LYL, WDY
S6	Fruiting body	PRJNA615295	GAC, LYL, WDY
F3	Fruiting body	PRJNA615295	GAC, LYL, WDY
F7	Fruiting body	PRJNA615295	GAC, LYL, WDY
F8	Fruiting body	PRJNA615295	GAC, LYL, WDY

Table S8. Locations of centromeric loci on each of the 14 *T. camphoratus* W1 chromosomes

Chromosome	Location (length (bp))
I	2685077-2784436 (99,360)
II	343472-404443 (60,972)
III	2034538-2115974 (81,437)
IV	1376462-1500537 (124,076)
V	1754025-1890826 (136,802)
VI	705104-780531 (75,428)
VII	1734833-1828147 (93,315)
VIII	57133-116031(58,899)
IX	1490128-1575402 (85,274)
X	612314-672038 (59,725)
XI	897544-975816 (78,272)
XII	971810-1092399 (120590)
XIII	214130-281339 (67,210)
XIV	564551-638577 (74,007)

Table S9. List of *HD1, HD2, mfa* and s*te3* genes in different *T. camphoratus* monokaryons.

Mating-type genes		W1	W2	V5	V7	s27 (1)
HD1	*HD1-1*					+
	HD1-2	+				
	HD1-3			+		
	HD1-4		+		+	
HD2	*HD2-1*		+		+	
	HD2-2	+				
	HD2-3					+
	HD2-4			+		
mfa	*mfa-1*	+	+	+	+	not determined
	mfa-2	+	+	+	+	
	mfa-3	+	+	+	+	
	mfa-4	+	+	+	+	
	mfa-5	-	-	+	-	
	mfa-6	-	-	+	-	
	mfa-7	-	-	+	-	
	mfa-8	-	-	+	-	
	mfa-9	+	+	+	+	
	mfa-10	+	+	-	+	
	mfa-11	-	-	-	+	
	mfa-12	-	+	-	-	
	mfa-13	-	+	-	-	
	mfa-14	+	-	-	-	
	mfa-15	+	-	-	-	
	mfa-16	+	+	-	+	
ste3	*Ste3*-1	+	+	-	-	not determined
	Ste3-2	+	+	-	-	
	Ste3-3	+	+	-	-	
	Ste3-4	+	-	-	+	
	Ste3-5	+	+	-	-	
	Ste3-6	+	+	-	-	
	Ste3-7	+	-	-	-	
	Ste3-8	+	-	-	-	
	Ste3-9	-	+	-	-	
	Ste3-10	-	+	-	-	
	Ste3-11	-	-	+	-	

	Ste3-12	-	-	+	-	
	Ste3-13	-	-	+	-	
	Ste3-14	-	-	+	-	
	Ste3-15	-	-	+	-	
	Ste3-16	-	-	-	+	
	Ste3-17	-	-	-	+	
	Ste3-18	-	-	-	+	
	Ste3-19	-	-	-	+	
	Ste3-20	-	-	-	+	
	Ste3-21	-	-	-	+	
	Ste3-22	-	-	-	+	

Table S10. Evolutionarily conserved fruiting-related genes (FRGs) in *T. camphoratus* or *Antrodia cinnamomea* (Ac)

FRG	Reference	Gene IDs (=protein IDs) of putative homolog(s) (e-value≦1e-17)
bri1	(2)	AcW1_000515
bwc2	(3)	AcW1_006061
cag1	(4)	AcW1_000308, AcW1_000537, AcW1_000701, AcW1_001159, AcW1_002351, AcW1_002389, AcW1_003841, AcW1_003985, AcW1_005830, AcW1_006069, AcW1_006747, AcW1_006905, AcW1_007504, AcW1_009174, AcW1_009290, AcW1_009291, AcW1_009509
c2h2	(2)	AcW1_003986, AcW1_003990, AcW1_009027, AcW1_009410
tupa	(4)	AcW1_001095, AcW1_001282, AcW1_001700, AcW1_002733, AcW1_002953, AcW1_003260, AcW1_003774, AcW1_004680, AcW1_004874, AcW1_005111, AcW1_005352, AcW1_006108, AcW1_006194, AcW1_006241, AcW1_006314, AcW1_006908, AcW1_008346, AcW1_009584, AcW1_009785
cfs1	(5)	AcW1_000074, AcW1_003000, AcW1_007602, AcW1_010238
cgl1	(6)	AcW1_000020
dst1	(7)	AcW1_009279
dst2	(8)	AcW1_003384, AcW1_007293
eln2	(9)	AcW1_000043, AcW1_000076, AcW1_000246, AcW1_000745, AcW1_001797, AcW1_001800, AcW1_002035, AcW1_002223, AcW1_002343, AcW1_002367, AcW1_002368, AcW1_002537, AcW1_002551, AcW1_003214, AcW1_003221, AcW1_005057, AcW1_005066, AcW1_005116, AcW1_005401, AcW1_005402, AcW1_005559, AcW1_006334, AcW1_007766, AcW1_007802, AcW1_009933, AcW1_009934
eln3	(10)	AcW1_002951, AcW1_009846, AcW1_010030
exp1	(11)	AcW1_004951
fst3	(2)	AcW1_003252, AcW1_005478, AcW1_005845, AcW1_006243, AcW1_008327, AcW1_008960, AcW1_009338

fst4	(2)	AcW1_000999, AcW1_001000, AcW1_002704, AcW1_002705, AcW1_003772, AcW1_003773, AcW1_003775, AcW1_006498, AcW1_006560, AcW1_006561, AcW1_006802, AcW1_006808, AcW1_007452
gat1	(2)	AcW1_004768, AcW1_007068
hemolysin	(12)	AcW1_000167, AcW1_003087
hom1	(2)	AcW1_009031
hom2	(2)	AcW1_009239
Ich1	(13)	AcW1_000566, AcW1_001402, AcW1_002535, AcW1_004349, AcW1_006336, AcW1_007286, AcW1_007287, AcW1_007303, AcW1_008887, AcW1_008914, AcW1_008915, AcW1_008946, AcW1_009665, AcW1_009688, AcW1_010153
lcc17	(14)	AcW1_001864, AcW1_002746, AcW1_006377, AcW1_006378, AcW1_006565, AcW1_006623, AcW1_007990
nsdD1	(12)	AcW1_008277
pcc1	(15)	AcW1_003718, AcW1_009620
velvet A-like	(12)	AcW1_002673, AcW1_006952
thaumatin-like	(12)	AcW1_002641, AcW1_002710

Table S11. Application of two different annotation methods to identify CAZymes in *T. camphoratus* and several other fungal model organisms

CAZyme families	AA	GH	GT	CE	CBM	PL	Total
Annotation methods	HMMER, DIAMOND or Hotpep						
	HMMER						
T. camphoratus W1	25	105	49	5	3	3	190
	34	118	64	31	3	4	254
T. camphoratus W2	27	112	48	7	5	3	202
	35	133	61	13	3	4	249
T. camphoratus V5	28	109	51	7	5	3	203
	35	129	63	12	3	4	246
T. camphoratus V7	25	111	49	7	6	2	200
	35	133	60	12	3	4	247
Ganoderma lucidum	85	185	46	11	21	7	355
	107	241	65	58	6	13	490
Pleurotus ostreatus	106	191	53	17	44	21	432
	139	227	67	27	29	26	515
Schizophyllum commune	56	173	61	18	97	16	421
	85	227	74	64	11	18	479

Table S12. List of gene numbers in different CAZyme subfamilies

Annotation method	HMMER						
Subfamilies	W1	W2	V5	V7	*G. lucidum*	*S. commune*	*P. ostreatus*
AA1	7	7	7	7	17	3	12
AA2	2	2	2	2	11	2	10
AA3	12	12	13	12	33	24	42
AA4	0	0	0	0	1	2	1
AA5	3	4	4	4	9	2	15
AA6	1	1	1	1	2	4	2
AA7	5	5	4	5	13	12	23
AA8	0	0	0	0	1	1	1
AA9	2	2	2	2	16	22	29
AA11	0	0	0	0	0	8	0
AA12	0	0	0	0	0	0	1
AA14	2	2	2	2	4	3	2
AA16	0	0	0	0	0	2	1
CBM1	0	0	0	0	0	0	4
CBM2	0	0	0	0	0	0	0
CBM3	0	0	0	0	0	0	0
CBM4	0	0	0	0	0	0	0
CBM5	0	0	0	0	1	0	0
CBM6	0	0	0	0	0	0	0
CBM10	0	0	0	0	0	0	0
CBM13	0	0	0	0	0	6	13
CBM18	0	0	0	0	0	0	0
CBM19	0	0	0	0	0	0	0
CBM20	1	1	1	1	2	1	3
CBM21	2	2	2	2	2	2	2
CBM22	0	0	0	0	0	0	0
CBM24	0	0	0	0	0	0	0
CBM25	0	0	0	0	0	0	0
CBM26	0	0	0	0	0	0	0
CBM27	0	0	0	0	0	0	0
CBM35	0	0	0	0	1	1	0
CBM42	0	0	0	0	0	0	0
CBM43	0	0	0	0	0	0	0
CBM48	0	0	0	0	0	0	0

CBM51	0	0	0	0	0	0	0
CBM54	0	0	0	0	0	0	0
CBM55	0	0	0	0	0	0	0
CBM56	0	0	0	0	0	0	0
CBM60	0	0	0	0	0	0	0
CBM63	0	0	0	0	0	1	0
CBM67	0	0	0	0	0	0	7
CE1	4	3	3	3	2	9	3
CE2	0	0	0	0	1	0	1
CE4	5	5	4	4	5	8	10
CE5	0	0	0	0	0	1	0
CE8	0	0	0	0	2	2	2
CE9	1	1	1	1	1	1	1
CE10	0	0	0	0	29	29	0
CE12	0	0	0	0	1	2	2
CE14	0	0	0	0	0	1	0
CE15	0	0	0	0	2	2	1
CE16	3	3	3	3	15	9	6
CE17	0	1	1	1	0	0	1
GH1	2	2	2	2	3	3	3
GH2	3	3	3	3	3	4	3
GH3	7	7	6	7	13	12	12
GH5	19	19	19	20	18	16	21
GH6	0	0	0	0	1	1	3
GH7	0	0	0	0	3	2	15
GH9	1	1	1	1	1	1	1
GH10	1	1	1	1	8	5	3
GH11	0	0	0	0	0	1	2
GH12	1	1	1	1	3	1	2
GH13	6	6	6	6	8	13	10
GH15	2	2	2	2	2	3	3
GH16	11	22	22	22	19	19	26
GH17	1	1	1	1	1	2	1
GH18	11	13	11	13	30	15	12
GH20	3	3	3	3	4	3	2
GH23	0	0	0	0	0	0	0
GH24	0	0	0	0	0	0	3

GH25	0	0	0	0	2	1	2
GH26	0	0	0	0	0	1	0
GH27	2	2	1	1	1	0	1
GH28	3	3	3	3	10	3	6
GH29	0	0	0	0	0	2	0
GH30	1	1	1	1	2	5	2
GH31	4	5	5	4	7	4	8
GH32	0	0	0	0	1	1	1
GH35	2	2	2	2	6	4	4
GH37	2	2	2	2	2	2	2
GH38	1	1	1	1	1	1	1
GH43	0	0	0	0	11	19	8
GH44	0	0	0	0	0	0	1
GH45	0	0	0	0	1	1	2
GH47	5	5	5	5	6	6	9
GH51	0	0	0	1	2	2	3
GH53	1	1	1	1	1	1	1
GH54	0	0	0	0	0	0	0
GH55	2	2	2	2	3	2	2
GH62	0	0	0	0	0	1	1
GH63	2	2	2	2	1	2	2
GH71	3	3	3	3	5	9	5
GH72	1	1	1	1	1	1	1
GH74	0	0	0	0	1	1	3
GH75	0	0	0	0	0	0	0
GH76	0	0	0	0	2	6	3
GH78	1	1	1	1	3	3	2
GH79	3	3	3	3	12	2	7
GH81	0	0	0	0	0	1	0
GH85	1	1	1	1	1	1	1
GH88	1	1	1	1	1	1	1
GH89	1	1	1	1	2	0	0
GH92	4	4	4	4	4	5	6
GH93	0	0	0	0	1	2	0
GH94	0	0	0	0	0	0	0
GH95	1	2	2	2	2	1	1
GH105	1	1	1	1	4	8	4

GH109	0	0	0	0	0	1	2
GH114	0	0	0	0	0	1	0
GH115	1	1	1	1	2	2	1
GH125	1	1	1	1	1	1	1
GH127	0	0	0	0	2	0	0
GH128	3	3	3	3	7	5	4
GH131	0	0	0	0	3	2	2
GH133	0	0	0	0	0	0	0
GH135	0	0	0	0	1	0	0
GH140	0	0	0	0	0	1	0
GH145	0	0	0	0	1	1	0
GH146	0	0	0	0	0	2	0
GH152	2	2	2	2	10	10	3
GH154	1	1	1	1	1	1	1
GH162	0	0	0	0	0	0	1
GT1	1	1	1	1	4	6	1
GT2	15	15	16	15	17	15	18
GT3	1	1	1	1	1	1	1
GT4	4	4	4	4	4	4	4
GT5	0	0	0	0	0	0	0
GT8	5	4	5	4	5	3	6
GT15	4	4	3	3	3	6	4
GT17	1	1	1	1	1	1	1
GT20	3	3	3	3	3	3	3
GT21	1	1	1	1	1	1	1
GT22	4	4	4	4	4	3	3
GT24	1	1	1	1	1	1	1
GT25	1	1	1	1	0	2	1
GT28	1	1	1	1	0	1	1
GT31	0	0	0	0	0	0	0
GT32	1	1	1	1	1	3	0
GT33	1	1	1	1	1	1	1
GT35	2	1	2	1	1	1	1
GT39	3	3	3	3	3	3	3
GT41	0	0	0	0	0	0	0
GT48	2	2	2	2	2	2	2
GT49	1	1	1	1	1	1	1

GT50	1	1	1	1	1	1	1
GT57	2	2	2	2	2	2	2
GT58	1	1	1	1	1	1	1
GT59	2	1	1	1	1	1	1
GT66	1	1	1	1	1	1	1
GT69	3	3	3	3	4	3	2
GT76	1	1	1	1	1	1	1
GT90	1	1	1	1	1	6	5
PL1	0	0	0	0	0	5	9
PL3	0	0	0	0	0	4	3
PL4	0	0	0	0	0	3	2
PL8	0	0	0	0	3	1	4
PL9	0	0	0	0	0	1	0
PL14	3	2	2	2	6	2	4
PL26	0	0	0	0	0	1	1
PL30	0	0	0	0	2	0	0
PL35	1	1	1	1	2	1	1
PL38	0	1	1	1	0	0	2

Table S13. Summary of the CAZ-GCs in the four *T. camphoratus* genomes

W1	W2	V5	V7
CAZ-GC_1.1	-	-	-
CAZ-GC_1.2	-	-	-
-	CAZ-GC_1.1	-	-
-	CAZ-GC_2.1	-	-
-	CAZ-GC_3.1	-	-
-	CAZ-GC_3.2	-	-
-	CAZ-GC_7.1	-	-
-	-	CAZ-GC_2.1	-
-	-	CAZ-GC_3.1	-
-	-	CAZ-GC_6.1	-
-	-	-	CAZ-GC_1.1
-	-	-	CAZ-GC_1.2
-	-	-	CAZ-GC_6.1
-	-	-	CAZ-GC_9.1
-	-	-	CAZ-GC_13.1
-	-	-	CAZ-GC_17.1

Table S14. Numbers of putative ergosterol biosynthetic genes in the genomes of the four *T. camphoratus* monokaryons

S. cerevisiae gene ID	*S. cerevisiae* gene name	W1	W2	V5	V7
YGR175C	*ERG1*	2	2	1	1
YMR202W	*ERG2*	1	1	1	1
YLR056W	*ERG3*	1	1	1	1
YGL012W	*ERG4*	1	1	1	1
YMR015C	*ERG5*	28	28	26	26
YML008C	*ERG6*	11	11	10	10
YHR072W	*ERG7*	1	1	1	1
YMR220W	*ERG8*	1	1	1	1
YHR190W	*ERG9*	1	1	1	1
YPL028W	*ERG10*	1	1	1	1
YHR007C	*ERG11*	26	28	28	28
YML126C	*ERG13*	1	1	1	1
YJL167W	*ERG20*	2	2	2	2
YNL280C	*ERG24*	2	4	3	3
YGR060W	*ERG25*	1	1	1	1
YGL001C	*ERG26*	4	4	4	4
YLR100W	*ERG27*	1	1	1	1
YER044C	*ERG28*	0	1	0	0

Table S15. Numbers of putative CYP genes

in the genomes of four *T. camphoratus* monokaryons and three model fungi

Class	s27[1]	s27[2]	W1	W2	V5	V7	*G. lucidum*[3]	*S. commune* H4-8[3]	*P. ostreatus* PC9[3]
CYP51	1	1	1	1	1	1	2	1	1
CYP53	2	2	2	2	2	2	1	1	3
CYP61	0	1	1	1	1	1	1	1	1
CYP63	6	8	7	7	7	7	6	8	7
CYP66	1	0	0	0	0	0	0	0	0
CYP502	0	2	2	1	2	1	1	0	3
CYP504	10	0	0	0	0	0	0	0	0
CYP505	5	0	0	0	0	0	4	1	1
CYP509	0	0	0	0	0	0	0	1	0
CYP512	6	6	6	6	6	6	22	7	5
CYP530	1	0	0	0	0	0	0	4	3
CYP537	1	1	1	1	1	1	1	0	0
CYP539	1	0	1	0	0	0	0	0	0
CYP609	1	1	1	1	1	1	0	0	0
CYP613	0	0	2	2	2	2	0	0	0
CYP619	1	0	0	0	0	0	0	3	2
CYP620	1	2	2	2	2	2	0	3	3
CYP621	0	1	0	0	0	0	0	9	1
CYP642	0	0	0	0	0	0	1	0	0
CYP645	2	0	1	1	1	1	0	0	0
CYP665	0	1	1	1	1	1	0	0	5
CYP684	0	0	0	0	0	0	0	2	0
CYP686	0	2	0	0	0	0	0	0	0
CYP5025	0	0	0	0	0	0	0	1	0
CYP5027	0	0	0	0	0	0	0	0	2
CYP5032	0	0	0	0	0	0	0	1	1
CYP5035	1	1	1	1	2	1	16	2	2
CYP5036	0	0	0	0	0	0	0	2	0
CYP5037	6	4	5	5	4	6	6	11	7
CYP5053	1	0	0	0	0	0	0	0	0
CYP5065	0	2	2	2	2	2	1	0	2
CYP5070	0	0	0	0	0	0	0	1	0
CYP5082	1	0	0	0	0	0	0	0	0
CYP5136	3	4	4	4	4	4	7	0	8

CYP5137	0	1	1	1	1	1	1	5	6
CYP5138	1	0	1	1	1	1	1	0	0
CYP5139	3	3	2	3	3	3	7	8	7
CYP5140	3	3	3	3	3	3	1	1	1
CYP5141	1	1	1	1	1	1	2	2	4
CYP5142	0	0	0	0	0	0	0	4	1
CYP5144	6	9	8	7	9	7	3	17	34
CYP5145	1	1	0	0	0	0	0	0	0
CYP5146	2	1	1	2	2	1	0	0	15
CYP5147	0	0	0	0	0	0	0	0	2
CYP5148	0	0	0	0	0	0	2	0	7
CYP5149	1	0	0	0	0	0	0	0	1
CYP5150	12	12	8	10	11	11	36	10	3
CYP5151	2	1	1	1	1	1	1	2	2
CYP5152	1	2	1	1	1	1	1	2	1
CYP5153	1	0	0	0	0	0	0	0	0
CYP5154	2	2	2	2	2	2	0	0	0
CYP5155	0	1	1	1	1	1	0	0	0
CYP5156	1	1	1	1	1	1	1	1	1
CYP5157	1	1	1	1	1	1	0	0	1
CYP5158	2	3	2	2	2	3	1	0	1
CYP5159	0	0	0	0	0	0	0	1	0
CYP5205	2	0	0	0	0	0	0	0	0
CYP5222	0	0	0	0	0	0	0	0	1
CYP5293	1	0	0	0	0	0	0	0	0
CYP5340	0	0	0	0	0	0	2	0	0
CYP5341	0	0	0	0	0	0	2	0	0
CYP5347	0	0	0	0	0	0	1	0	0
CYP5348	0	0	0	0	0	0	3	0	0
CYP5349	0	0	0	0	0	0	1	0	0
CYP5351	0	0	0	0	0	0	1	0	0
CYP5357	0	0	0	0	0	0	2	0	0
CYP5358	0	0	0	0	0	0	1	0	0
CYP5359	0	0	0	0	0	0	47	0	0
CYP5360	0	0	0	0	0	0	1	0	0
CYP5361	0	0	0	0	0	0	1	0	0
CYP5362	0	0	0	0	0	0	1	0	0

CYP5363	0	0	0	0	0	0	1	0	0
CYP5364	0	0	0	0	0	0	3	0	0
CYP5365	0	0	0	0	0	0	1	0	0
CYP5366	0	0	0	0	0	0	1	0	0
CYP6001	1	0	0	0	0	0	0	1	4
CYP6004	1	0	0	0	0	0	0	1	0
CYP6005	0	0	0	0	0	0	2	0	0

[1]From the original s27 genome annotation (1).

[2]From the genome annotation by Funannotate (this study).

[3]From Fungal Cytochrome P450 Database (http://p450.riceblast.snu.ac.kr/intro.php).

Table S16. Summary of the SM-BGCs in the four *T. camphoratus* haploid genomes

W1	W2	V5	V7
BGC_1	BGC_1	BGC_1	BGC_1
BGC_2	-	-	BGC_2
BGC_3	BGC_3	BGC_3	BGC_3
BGC_4	BGC_4	BGC_4	BGC_4
BGC_5	BGC_5	BGC_5	BGC_5
BGC_6	BGC_6	BGC_6	BGC_6
BGC_7	BGC_7	BGC_7	BGC_7
BGC_8	BGC_8	BGC_8	BGC_8
BGC_9	BGC_9	BGC_9	BGC_9
BGC_10	BGC_10	BGC_10	BGC_10
BGC_11	BGC_11	BGC_11	BGC_11
BGC_12	BGC_12	BGC_12	BGC_12
BGC_13	BGC_13	BGC_13	BGC_13
BGC_14	BGC_14	BGC_14	BGC_14
BGC_15	BGC_15	BGC_15	BGC_15
BGC_16	BGC_16	BGC_16	BGC_16
BGC_17	BGC_17	BGC_17	BGC_17
BGC_18	BGC_18	BGC_18	BGC_18
BGC_19	BGC_19	BGC_19	BGC_19
BGC_20	BGC_20	BGC_20	BGC_20
BGC_21	BGC_21	BGC_21	BGC_21
BGC_22	BGC_22	BGC_22	BGC_22
BGC_23	BGC_23	BGC_23	BGC_23
BGC_24	BGC_24	BGC_24	BGC_24
BGC_25	BGC_25	BGC_25	BGC_25
BGC_26	BGC_26	BGC_26	BGC_26
-	-	BGC_27	-

Table S17. Thirty-seven transcriptionally upregulated genes in the orange-red strains of *T. camphoratus* or *Antrodia cinnamomea* (Ac)

W1 Gene ID	*Neurospora* Gene ID	*Neurospora* Gene Name	*Neurospora* Gene Description	SGD Gene ID	SGD Gene Name
AcW1_005073	NCU06351T0	*pht-1*	phytase-1	YDL024C	*DIA3*
AcW1_005074	NCU07308T0	*cel-1*	-	YPL231W	*FAS2*
AcW1_005075	NCU07308T0	*cel-1*	-	YPL231W	*FAS2*
AcW1_005077	-		-	-	-
AcW1_010300	-		-	-	-
AcW1_005078	NCU08132T0	*ags-2*	alpha-1,3-glucansynthase Ags2	-	-
AcW1_010301	-		-	-	-
AcW1_005080	-		-	-	-
AcW1_010302	-		-	-	-
AcW1_005084	NCU08132T0	*ags-2*	alpha-1,3-glucansynthase Ags2	-	-
AcW1_005085	-		-	-	-
AcW1_010305	-		-	-	-
AcW1_005091	NCU08132T0	*ags-2*	alpha-1,3-glucansynthase Ags2	-	-
AcW1_005227	NCU06009T0		oxidoreductase	YPL088W	-
AcW1_005230	NCU06009T0		oxidoreductase	YPL088W	-
AcW1_005398	-		-	-	-
AcW1_005559	NCU01134T0	*cyp450-4*	hypothetical protein	YMR015C	*ERG5*
AcW1_005848	NCU07308T0	*cel-1*	-	YPL231W	*FAS2*
AcW1_005849	NCU07308T0		-	YPL231W	*FAS2*
AcW1_006854	NCU07308T0		-	YPL231W	*FAS2*
AcW1_007136	NCU02056T0		salicylaldehyde dehydrogenase	YER073W	*ALD5*
AcW1_007228	NCU07959T0	*pho-13*	hypothetical protein	-	-
AcW1_007239	-		-	-	-
AcW1_008584	NCU02888T0		hypothetical protein	-	-
AcW1_008667	-		-	-	-
AcW1_009423	-		-	-	-
AcW1_009761	NCU01839T0	*cea-7*	carboxyl esterase	-	-
AcW1_002053	NCU07240T0	aflatoxin B1 aldehyde reductase member 2	-	YDL243C	*AAD4*
AcW1_002142	-		-	-	-
AcW1_002143	-		-	-	-
AcW1_002440	NCU05162T0		hypothetical protein	YER080W	*AIM9*
AcW1_010269	-		-	-	-
AcW1_002896	NCU09519T0	*ara-1*	2,5-diketo-D-gluconic acid reductase A	YOR120W	*GCY1*
AcW1_002948	-		-	-	-
AcW1_003255	-		-	-	-
AcW1_003407	-		-	-	-

AcW1_003581	-		-	-	-

Table S18. Putative secondary metabolite biosynthetic genes

N. crassa	*C. cinerea*	*G. lucidum*	*S. commune*	W1	W2	V5	V7	s27[1]	s27[2]
NCU07008<br>*cao-1*	-	-	-	-	-	-	-	-	-
NCU11424<br>*cao-2*	-	-	-	000042	001464	003937	007097	006935	005325
NCU00585<br>*al-1*	-	-	-	-	-	-	-	-	-
NCU00552<br>*al-2*	-	-	-	-	-	-	-	-	-
NCU04013<br>*ylo-1*	EAU89477	53863<br>121149	61827<br>79669	005500	004268	008852	009188	010013	007732
NCU04452<br>EasA/FgaOx3	EAU87267	45271	70977	010050	002175	002945	007185	009220	007077
NCU12075<br>FgaPT1	EAU82200	-	78007	009878	002335	003285	005231	011507	008849
NCU04343<br>EasF	EAU85926	116943	67573	007327	007602	007967	006035	007486	005730
NCU07619<br>EasE	EAU82888	123504	12964	001860	010187	000090	001711	001036	000776
NCU05169<br>EasC	EAU86801	86439	46891	009851	002364	003311	007014	011667	008952
NCU02128<br>EasD	EAU88723	119334	50261	006062	004824	006382	008687	-	-
NCU07474<br>(*eabA)*<br>ergot alkaloid biosynthetic protein A<br>EsaG	EAU83398	-	57029	-	-	-	-	-	-

[1]From the genome annotation by Funannotate (this study).

[2]From the original s27 genome annotation (1).

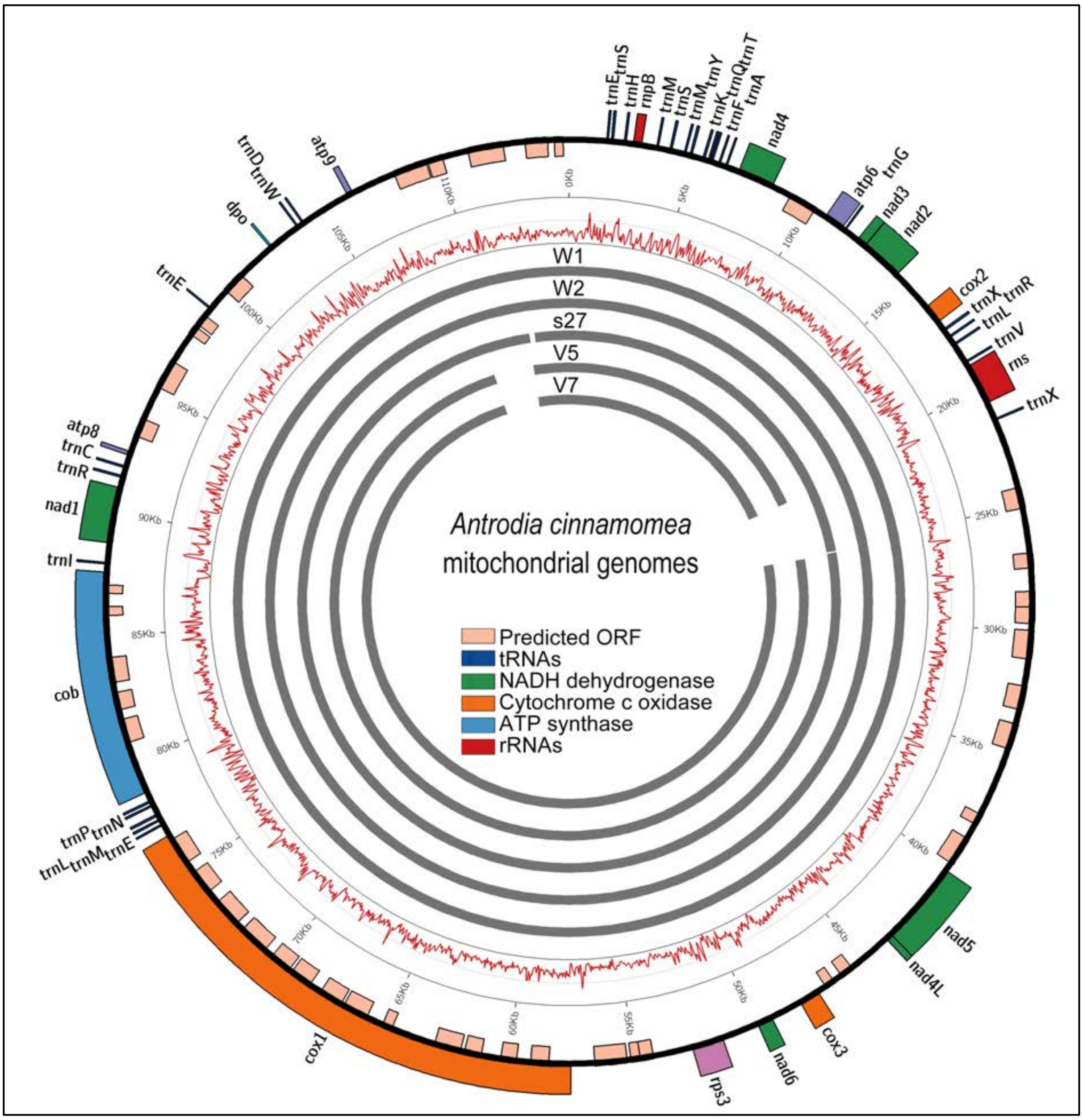

Figure S1. Circular map of *T. camphoratus* [*Antrodia cinnamomea* (*Ac*)] mitochondrial genomes, drawn using Organellar Genome Draw (OGDRAW) (16). Genes are represented by boxes, which are colored on the basis of their function. The second external plot shows the GC content of the W1 genome sequence, with the grey line marking 50%. The five internal plots show the nucleotide sequence alignment of five *T. camphoratus* genomes, as indicated. The detailed annotation of the mitochondrial genomes is shown in Dataset DS1.

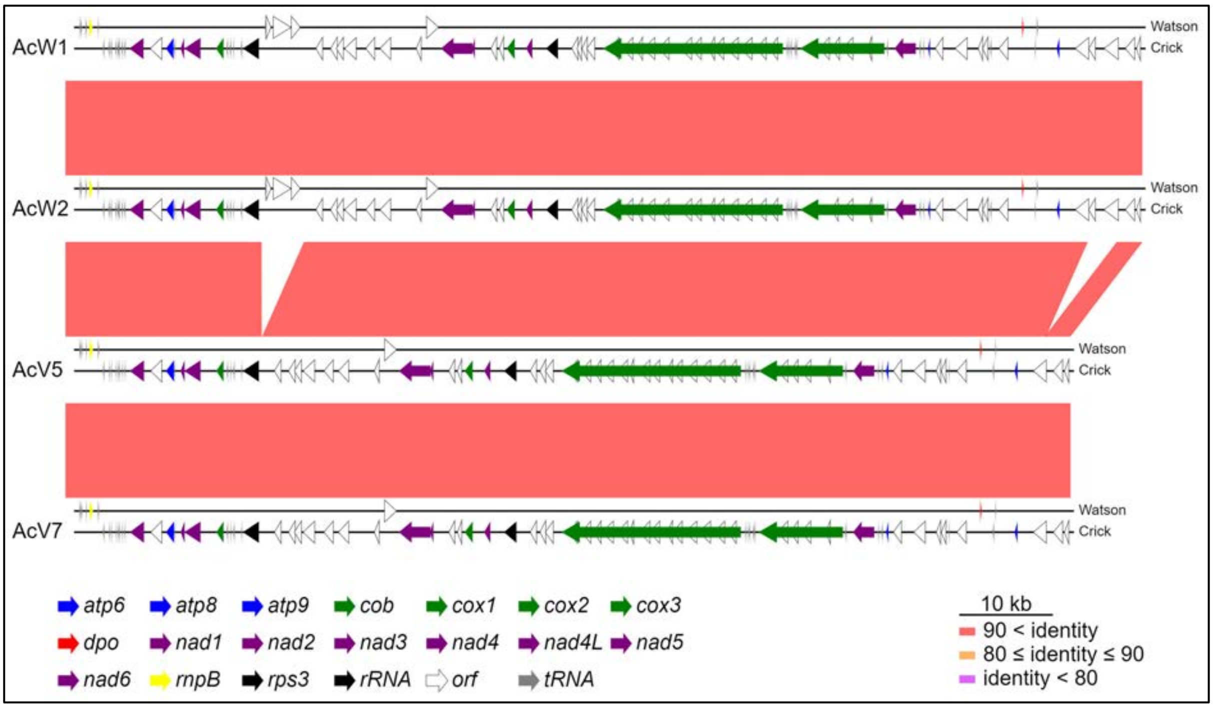

Figure S2. Collinearity relationship between the mitochondrial genome sequences of W1, W2, V5 and V7. The two DNA strands are indicated as W and C, respectively. The detailed annotation of the mitochondrial genomes is shown in Dataset DS1.

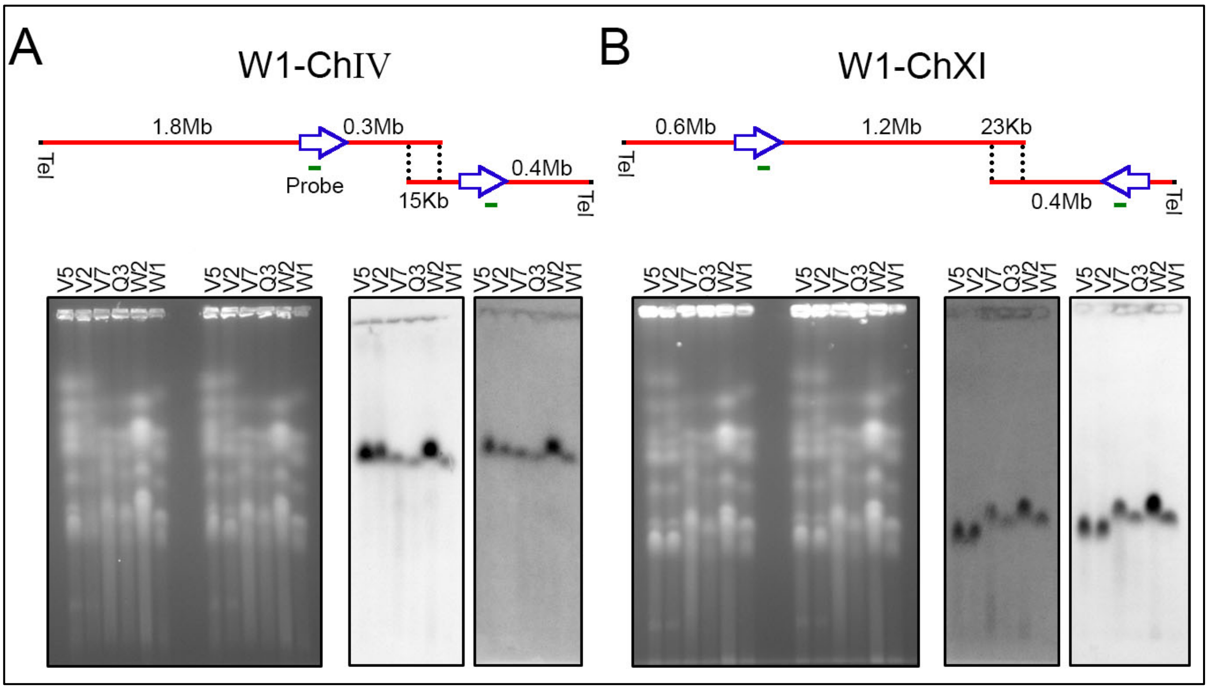

Figure S3. W1 Chromosome IV (W1-ChIV) and W1 chromosome XI (W1-ChXI) each consists of two overlapping superscaffords, i.e., ChIVA, ChIVB, ChXIA and ChXIB, respectively. PFGE was applied to separate the chromosomes of V5, V2, V7, Q3, W1 and W2. Blue arrows indicate the four DNA probes used for Southern hybridization. The overlapping regions between ChIVA and ChIVB (A) or between ChXIA and ChXIB (B) are indicated by two dotted lines, respectively.

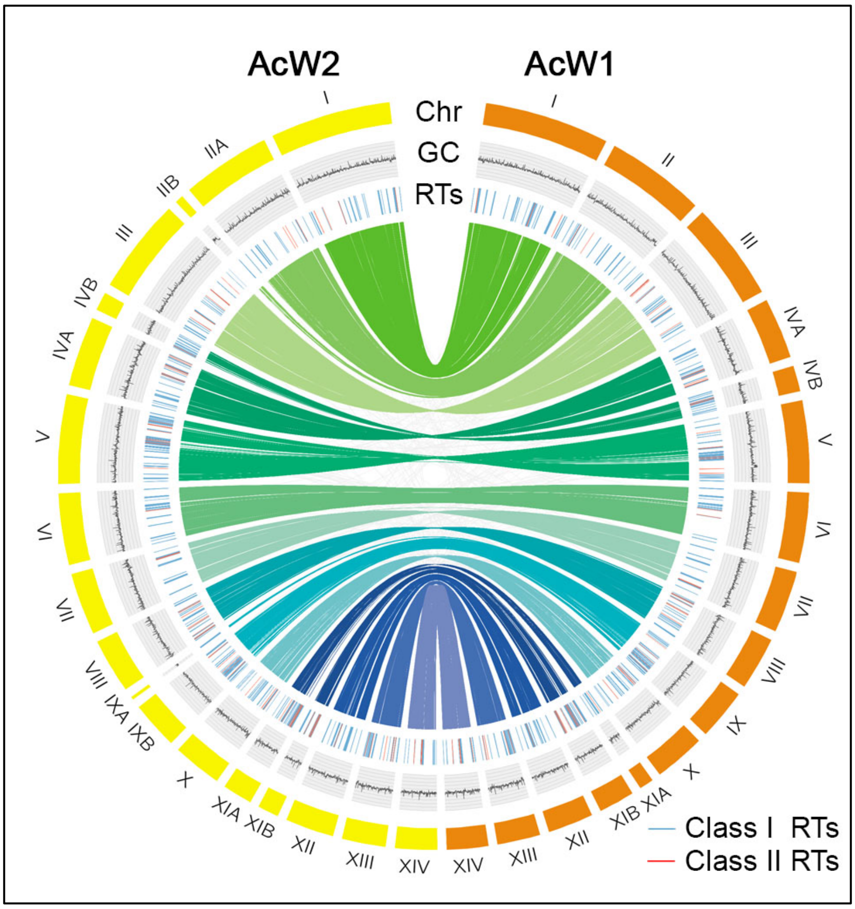

Figure S4. Synteny around the chromosomes of W1 (in orange) and W2 (in yellow). The middle plot shows the GC content. The internal plot shows the location of RTs. *Ac*: *Antrodia cinnamomea.*

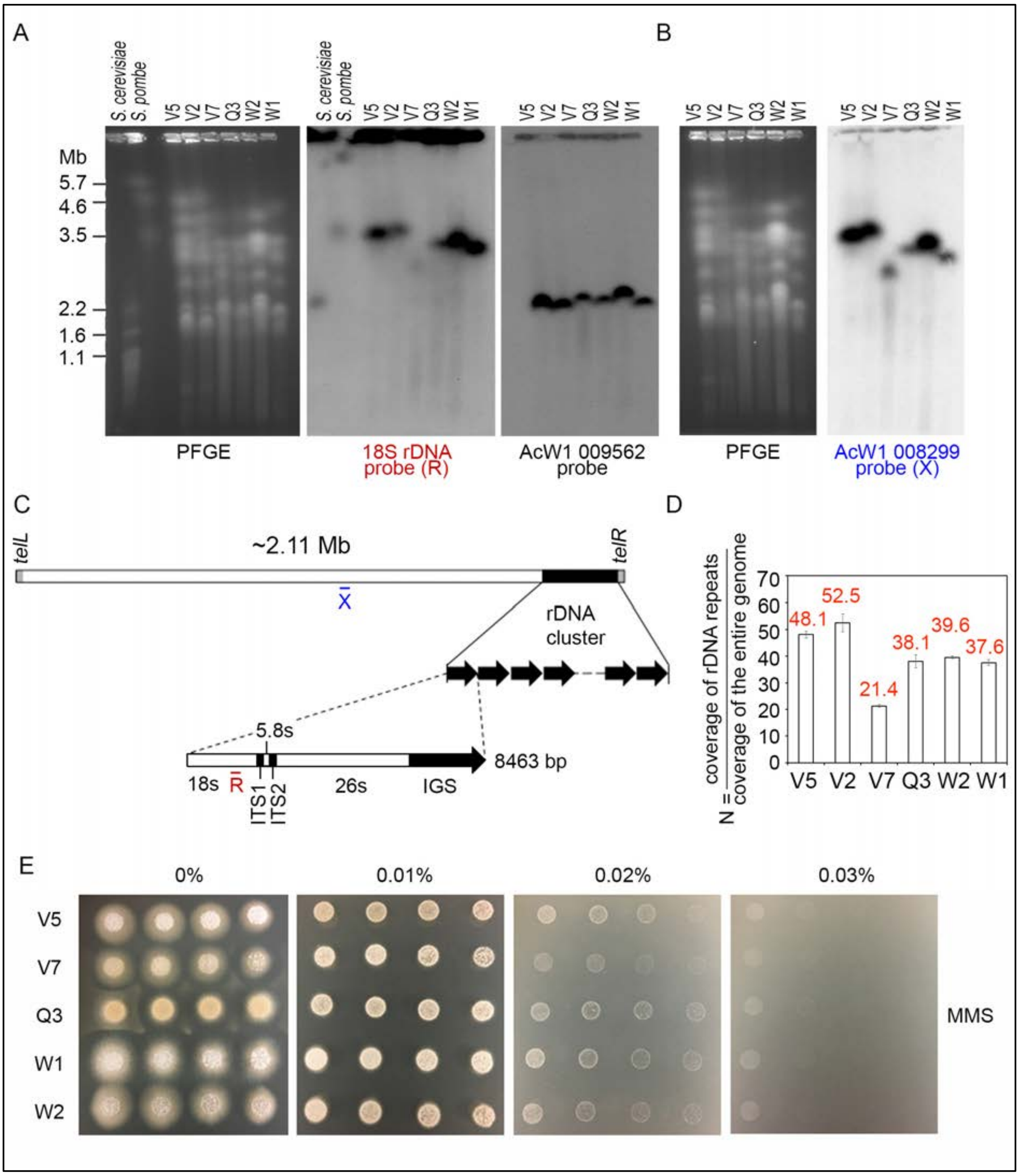

Figure S5. Copy number variations of the 45S ribosomal DNA loci in different monokaryons. (A and B) PFGE was applied to separate the chromosomes of *S. cerevisiae*, *S. pombe*, V5, V2, V7, Q3, W1 and W2. Southern hybridization with three DNA probes as indicated. (C) Organization of the rDNA locus. Illustration of W1-ChX and the nine tandem "head-to-tail" repeats revealed by the PacBio RSII platform. Each repeat contains an 18s-ITS1-5.8s-ITS2-26s rRNA gene cluster and a full-length non-transcribed intergenic spacer (IGS). ITS1 and ITS2 are two internal transcribed spacers. (D) The ratios (N; in red) of mapping coverage of Illumina MiSeq reads specifically for the rDNA locus versus those for the entire genomic DNA. (E) The arthospores of five different *T. camphoratus* monokaryons were collected from

vegetative cultures, quantified with a hemocytometer, and then germinated in PDB media for 16 hrs at room temperature, respectively. Spot assay showing five-fold serial dilutions of the newly germinated monokaryons grown on MEA plates without MMS or with MMS at the indicated concentrations (w/v).

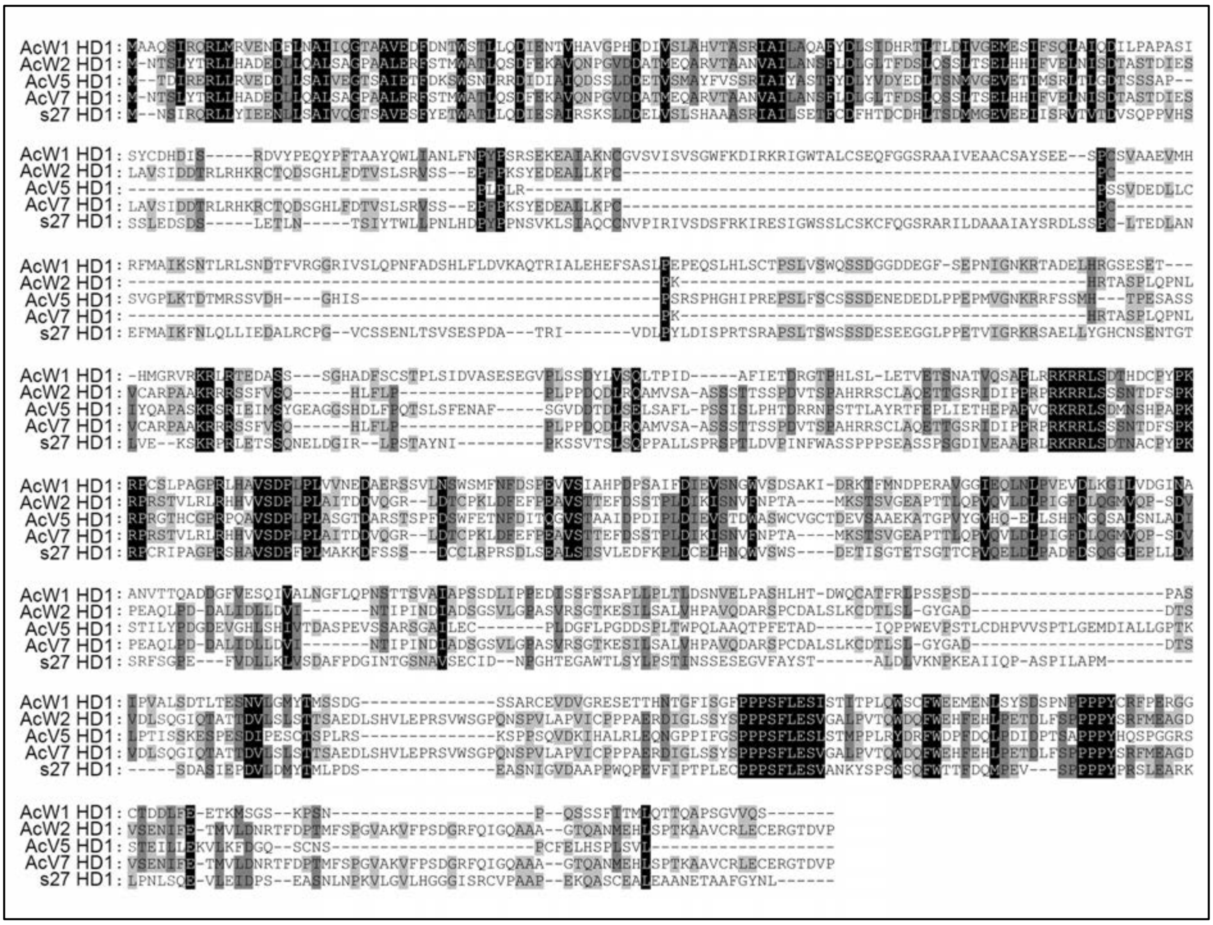

Figure S6. Amino acid sequence alignment of five HD1 proteins. Identical and similar residues are indicated with white letters in black and black letters in grey boxes, respectively. Dashes indicate the absence of residues. *Ac*: *Antrodia cinnamomea.*

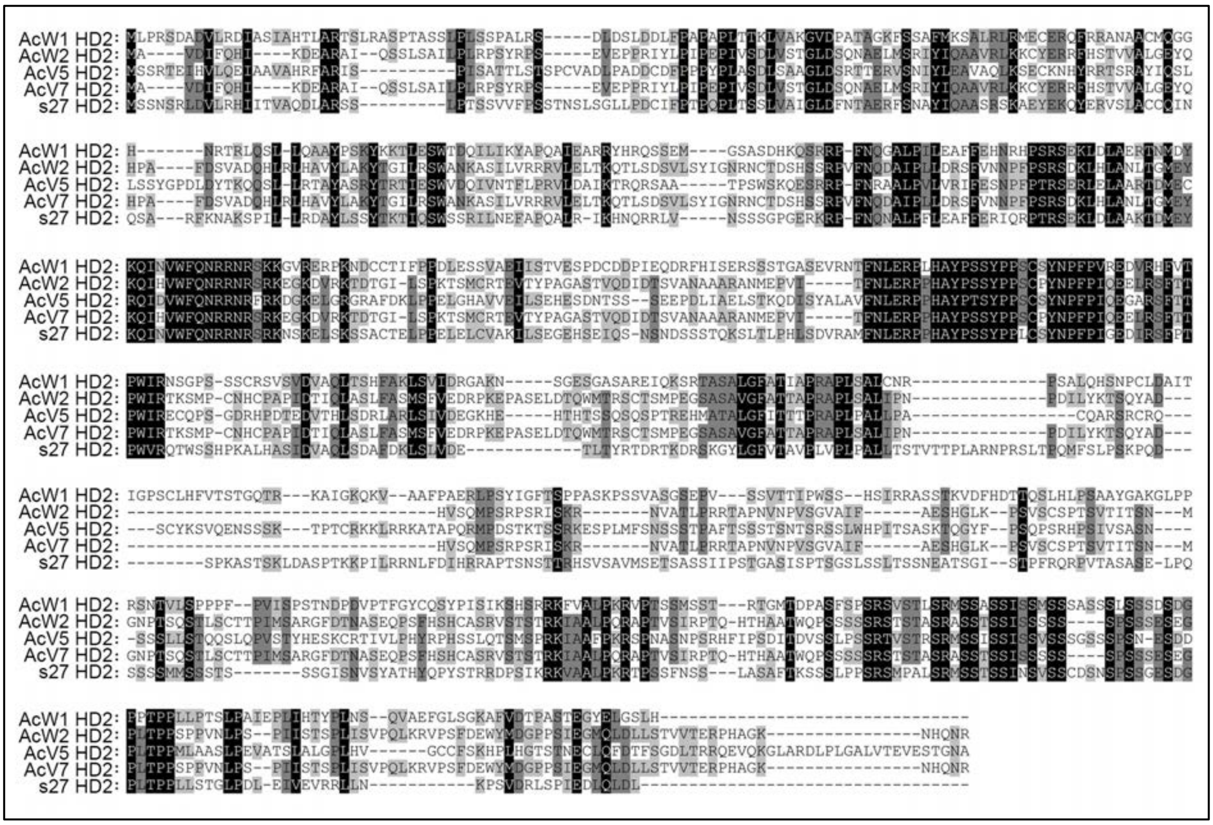

Figure S7. Amino acid sequence alignment of five HD2 proteins. Identical and similar residues are indicated with white letters in black and black letters in grey boxes, respectively. Dashes indicate the absence of residues. *Ac*: *Antrodia cinnamomea.*

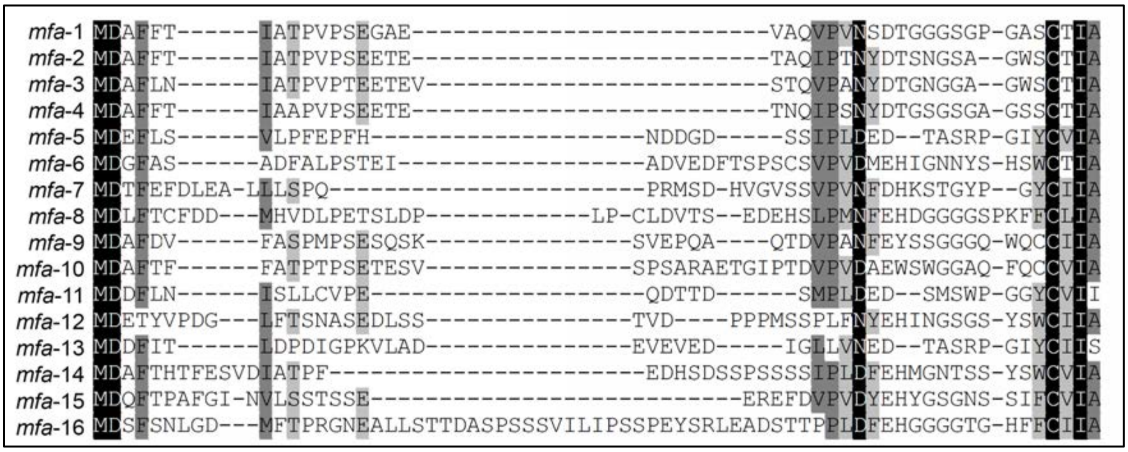

Figure S8. Amino acid sequence alignment of 17 mating pheromone proteins. Identical and similar residues are indicated with white letters in black and black letters in grey boxes, respectively. Dashes indicate the absence of residues.

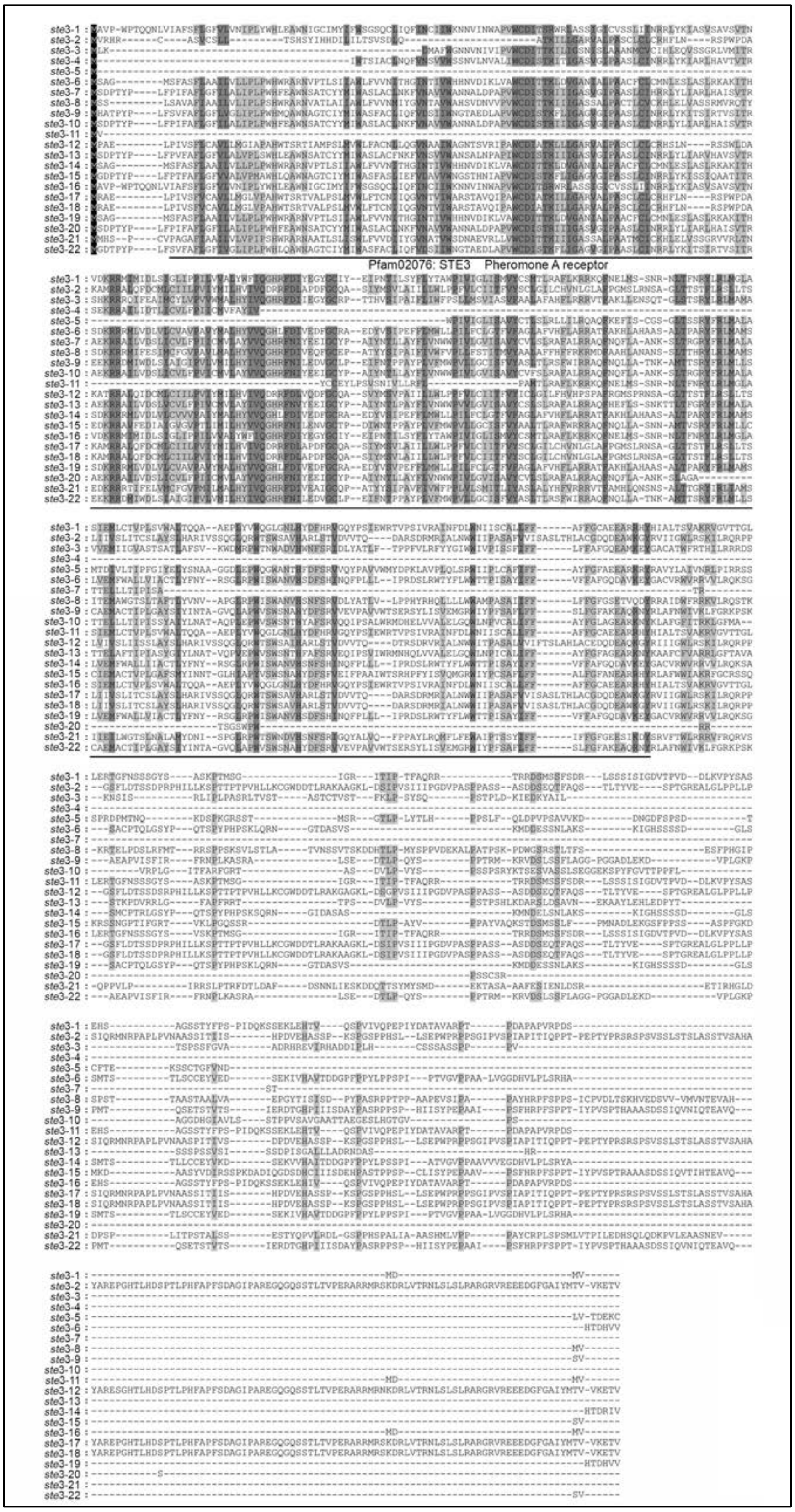

Figure S9. Amino acid sequence alignment of 22 mating pheromone receptor proteins. Identical and similar residues are indicated with white letters in black and black letters in grey boxes, respectively. Dashes indicate the absence of residues.

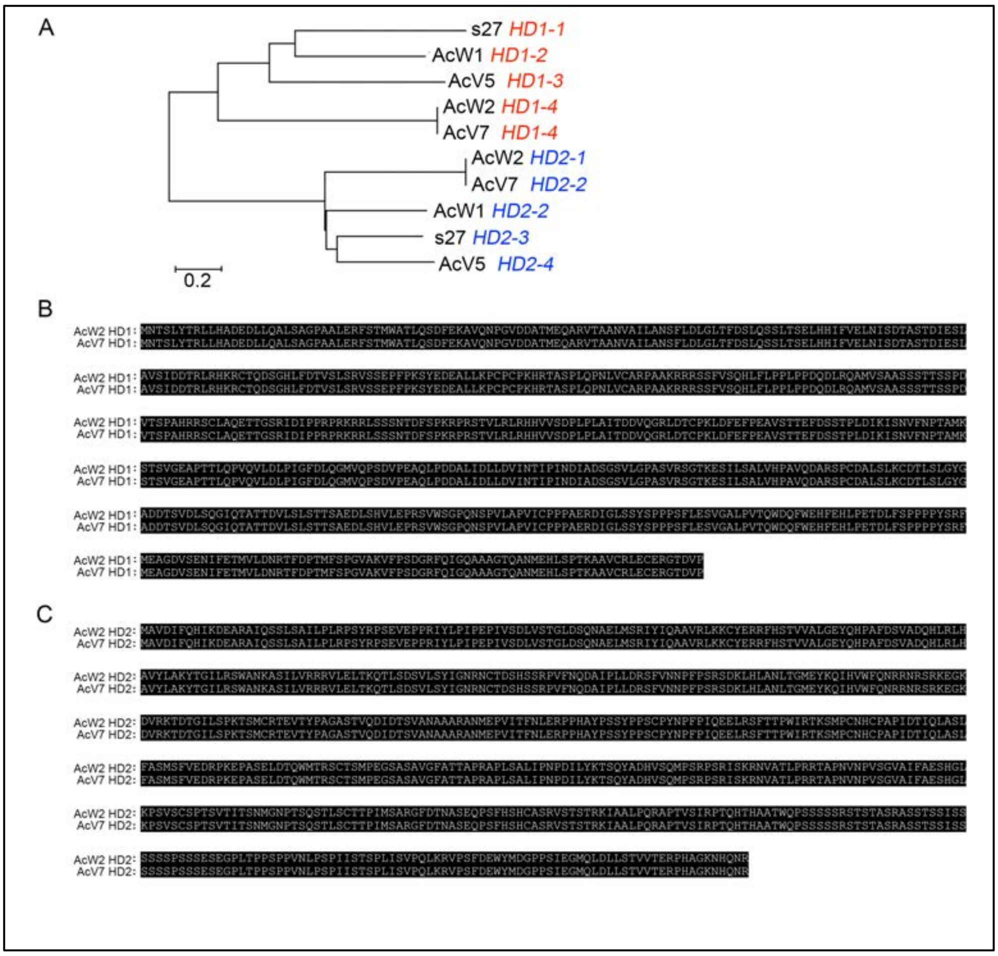

Figure S10. W2 and V7 share the same HD1 and HD2 proteins. (A) Phylogenetic tree of all five HD1 and five HD2 proteins. Evolutionary history was inferred by using a Maximum Likelihood approach and the JTT matrix-based model in MEGA7 (17). The tree is drawn to scale, with branch lengths representing the number of substitutions per site. (B and C) Amino acid sequence alignment of HD1 and HD2 proteins in W2 and V7. *Ac*: *Antrodia cinnamomea.*

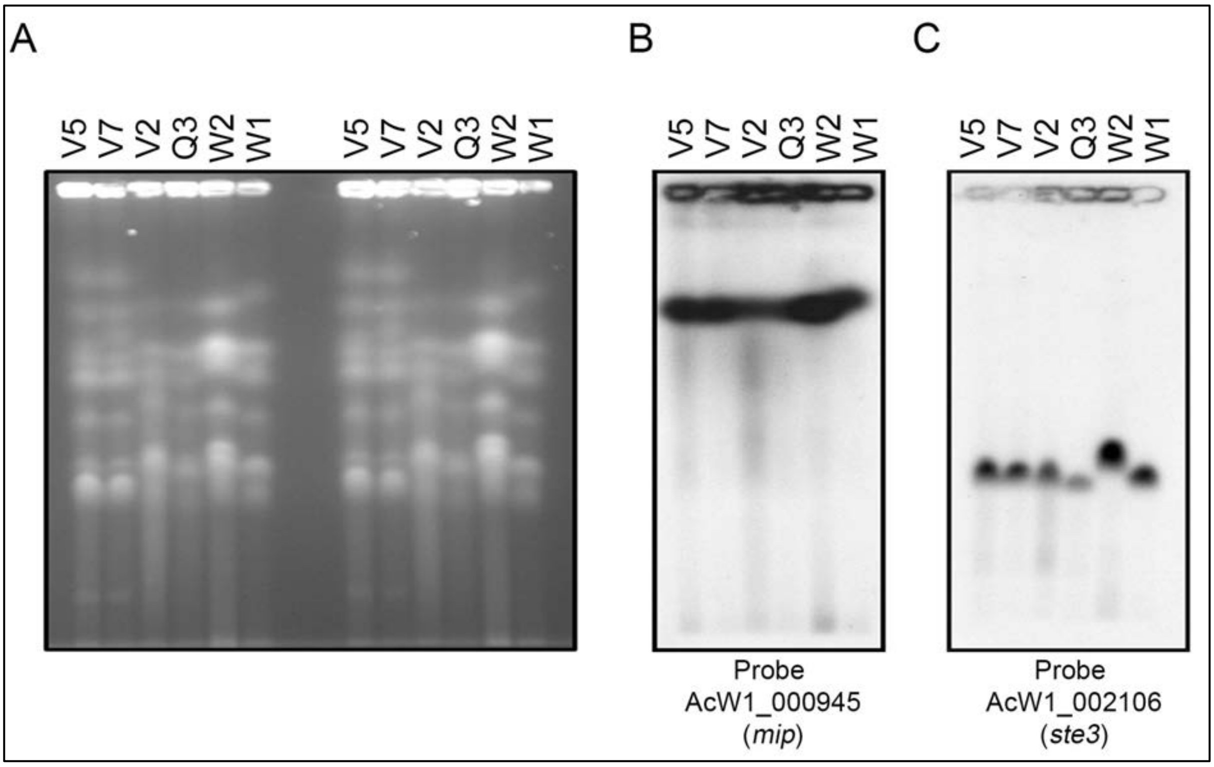

Figure S11. *Ac* has a tetrapolar mating system. (A) PFGE was applied to separate the chromosomes of V5, V2, V7, Q3, W1 and W2. (B-C) Southern hybridizations with two DNA probes, *mip* (AcW1_000945) and *ste3* (AcW1_002106), are indicated, respectively.

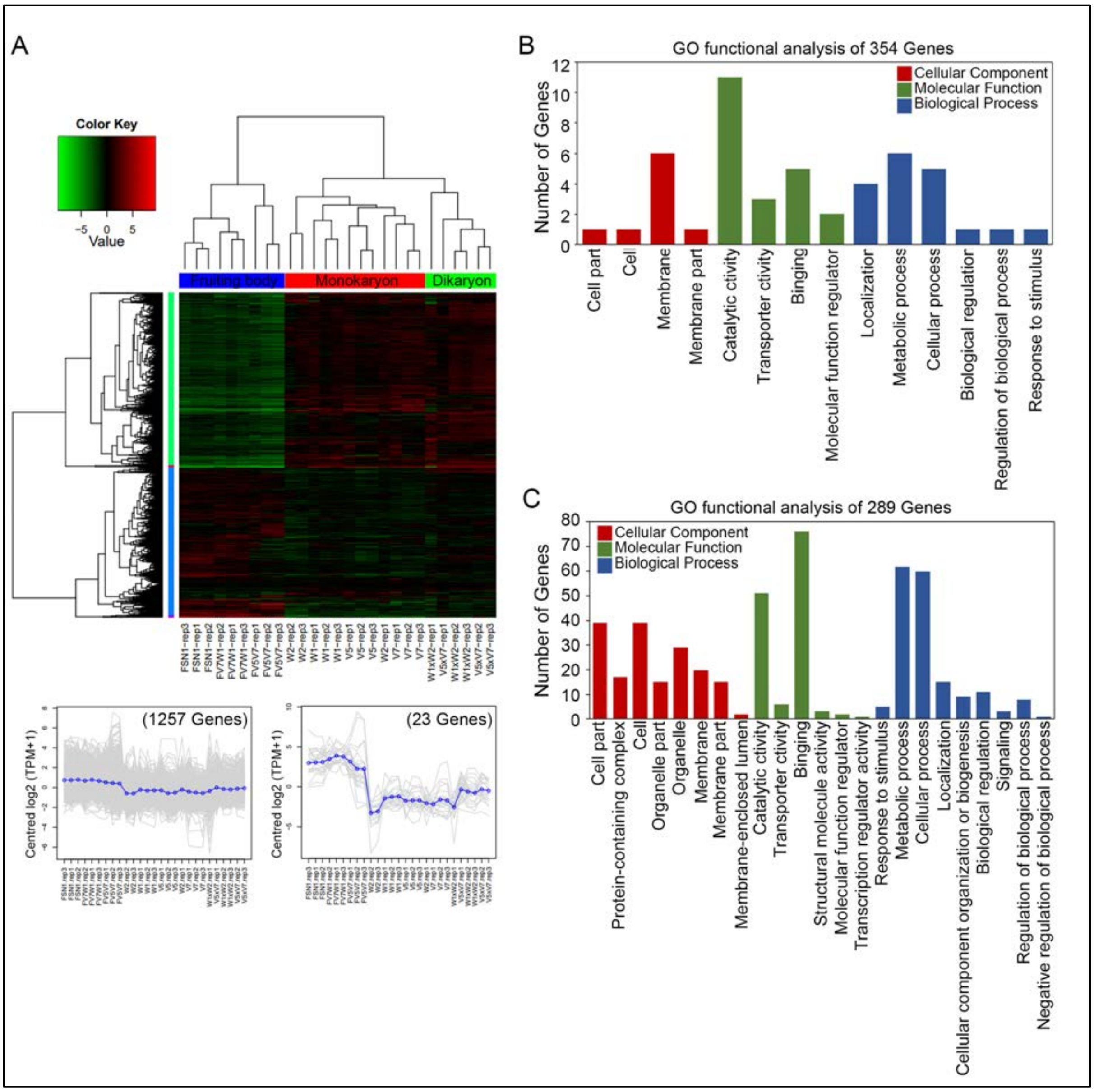

Figure S12. Identification of *Ac*FRGs. (A) Heatmap of the hierarchical cluster analysis showing centered log2 gene expression across all 28 RNA-seq datasets. Two subclusters were recovered from the heatmap of differentially upregulated protein-encoding genes for fruiting bodies versus vegetative mycelia of four monokaryons and two dikaryons. (B, C) Gene ontology (GO) annotation and enrichment analysis of 354 *Ac*-specific FRGs (Figure 5C) (B) and the 289 *Ac*-FRGs whose *C. cinerea* orthologs are neither *Cc*FBIGs nor *Cc*FBDGs (Figure 5C) (C). *Ac*: *Antrodia cinnamomea. Cc*: *C. cinereal.*

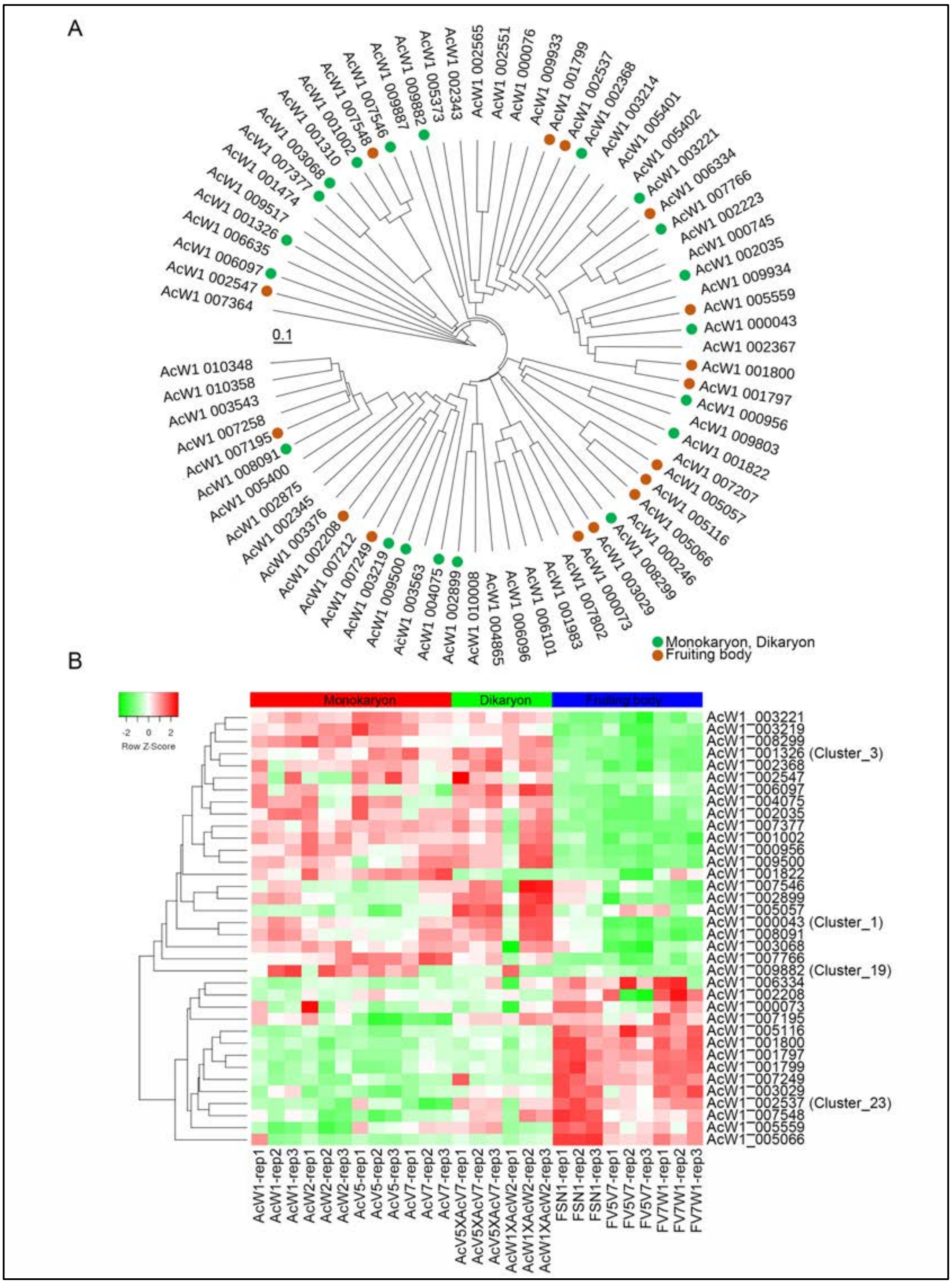

Figure S13. CYPs. (A) Circular phylogenetic tree depicting the relationships between amino acid sequences of all CYP450 proteins in W1. The CYP450 protein genes that are transcriptionally upregulated in fruiting bodies and vegetative mycelia of monokaryons and dikaryons are indicated by red and green circles, respectively. (B) Heatmap of the hierarchical cluster analysis showing centered log2 gene expression of the differentially expressed CYP genes across all 27 RNA-seq datasets. The CPY genes located in the SM-BGCs are indicated. *Ac*: *Antrodia cinnamomea.*

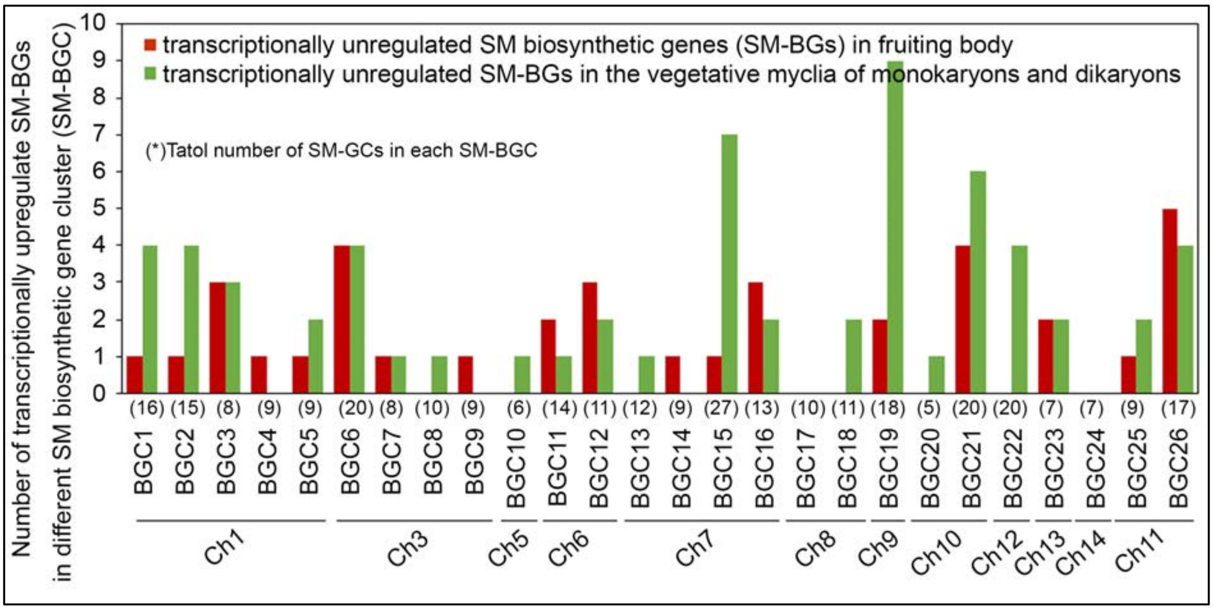

Figure S14. Transcriptional profiles of the 26 SM-BGCs in W1. Numbers of transcriptionally upregulated SM-BGs in fruiting bodies and in vegetative mycelia of monokaryons and dikaryons are indicated in red and green, respectively. Total number of SM-BGs in each SM-BGC is indicated in brackets.

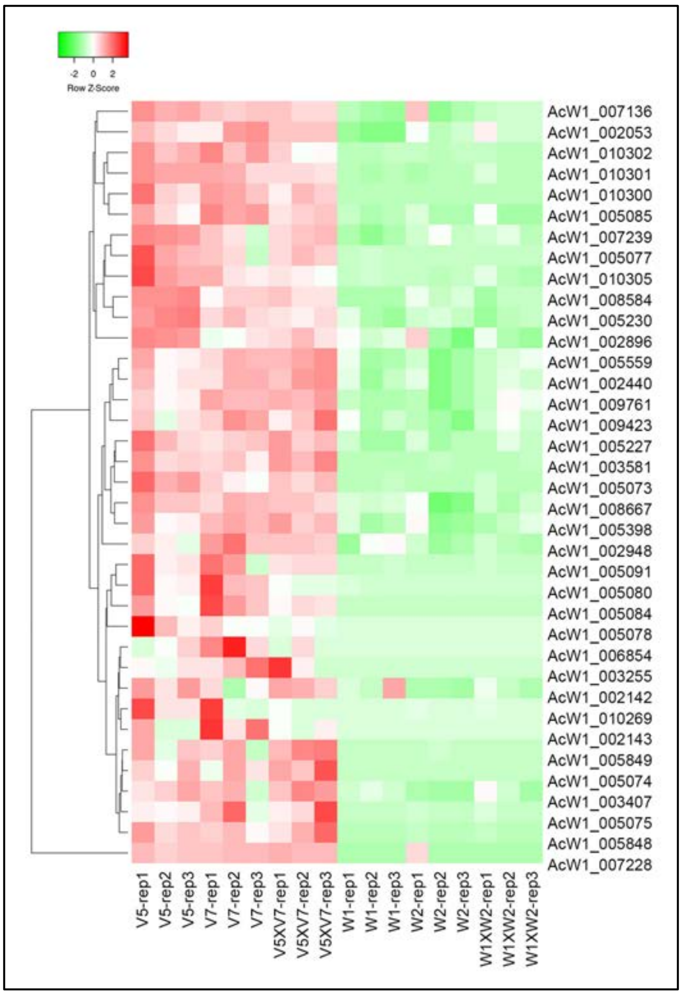

Figure S15. Identification of *T. camphoratus* genes that are transcriptionally upregulated in orange-red vegetative mycelia (V5, V7 and V5xV7) relative to milky-white vegetative mycelia (W1, W2 and W1xW2). Heatmap of the hierarchical cluster analysis showing centered log2 gene expression of the differentially expressed genes across all 18 RNA-seq datasets.

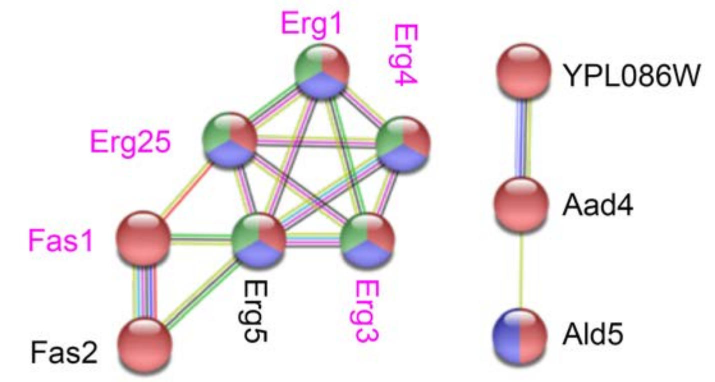

Biological Process (Gene Ontology)

GO-term	description	count in network	strength	false discovery rate
GO:0055114	oxidation-reduction process	11 of 457	1.09	1.12e-09
GO:0006066	alcohol metabolic process	7 of 93	1.59	1.01e-08
GO:0006696	ergosterol biosynthetic process	5 of 25	2.01	5.03e-08
GO:0008610	lipid biosynthetic process	7 of 178	1.31	1.03e-07
GO:0044283	small molecule biosynthetic process	8 of 345	1.08	2.86e-07

Figure S16. The STRING protein-protein interaction network queried with the *S. cerevisiae* orthologs of *T. camphoratus* proteins that are transcriptionally upregulated in orange-red vegetative mycelia (V5, V7 and V5xV7) relative to milky-white vegetative mycelia (W1, W2 and W1xW2). Colored lines between the yeast proteins indicate the various types of interaction evidence. In the bottom inset, three enriched functions have been selected, and the corresponding protein nodes in the network are automatically highlighted in color. The accessory information available for the reported enrichment of functional connections among the set of proteins and statistical enrichments detected in functional subsystems is also presented.

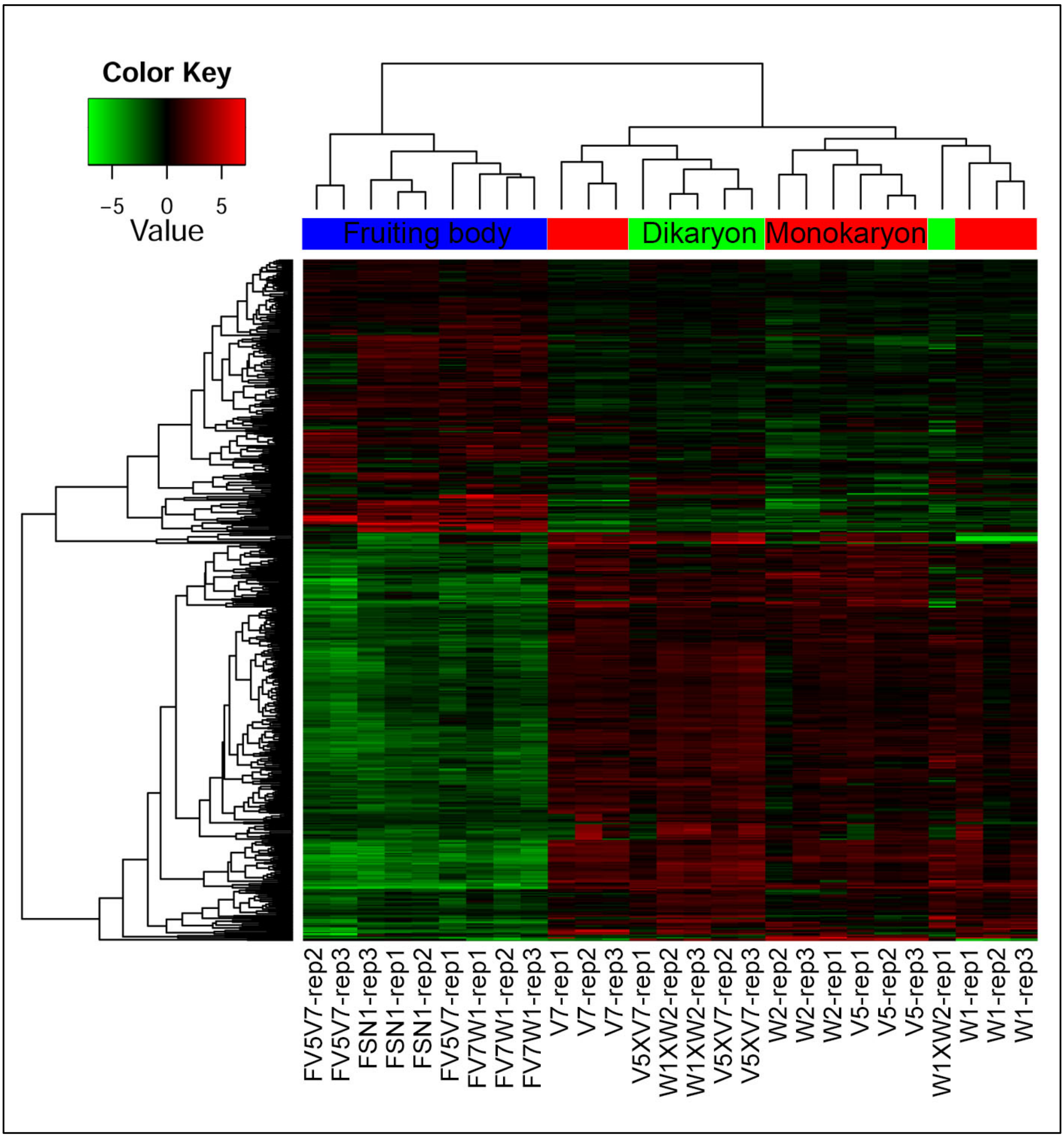

Figure S17. Identification of *T. camphoratus* lncRNA genes that are transcriptionally upregulated in vegetative mycelia and fruiting bodies. Heatmap of the hierarchical cluster analysis showing centered log2 gene expression of the differentially expressed genes across all 27 RNA-seq datasets.

References:

1. Lu, M.Y., Fan, W.L., Wang, W.F., Chen, T., Tang, Y.C., Chu, F.H., Chang, T.T., Wang, S.Y., Li, M.Y., Chen, Y.H. *et al.* (2014) Genomic and transcriptomic analyses of the medicinal fungus *Antrodia cinnamomea* for its metabolite biosynthesis and sexual development. *Proc Natl Acad Sci U S A*, **111**, E4743-4752.
2. Ohm, R.A., de Jong, J.F., de Bekker, C., Wosten, H.A. and Lugones, L.G. (2011) Transcription factor genes of *Schizophyllum commune* involved in regulation of mushroom formation. *Mol Microbiol*, **81**, 1433-1445.
3. Idnurm, A. and Heitman, J. (2005) Light controls growth and development via a conserved pathway in the fungal kingdom. *PLoS Biol*, **3**, e95.
4. Masuda, R., Iguchi, N., Tukuta, K., Nagoshi, T., Kemuriyama, K. and Muraguchi, H. (2016) The *Coprinopsis cinerea* Tup1 homologue Cag1 is required for gill formation during fruiting body morphogenesis. *Biol Open*, **5**, 1844-1852.
5. Liu, Y., Srivilai, P., Loos, S., Aebi, M. and Kues, U. (2006) An essential gene for fruiting body initiation in the basidiomycete *Coprinopsis cinerea* is homologous to bacterial cyclopropane fatty acid synthase genes. *Genetics*, **172**, 873-884.
6. Boulianne, R.P., Liu, Y., Aebi, M., Lu, B.C. and Kues, U. (2000) Fruiting body development in *Coprinus cinereus*: regulated expression of two galectins secreted by a non-classical pathway. *Microbiology (Reading)*, **146 (Pt 8)**, 1841-1853.
7. Terashima, K., Yuki, K., Muraguchi, H., Akiyama, M. and Kamada, T. (2005) The *dst1* gene involved in mushroom photomorphogenesis of *Coprinus cinereus* encodes a putative photoreceptor for blue light. *Genetics*, **171**, 101-108.
8. Kamada, T., Sano, H., Nakazawa, T. and Nakahori, K. (2010) Regulation of fruiting body photomorphogenesis *in Coprinopsis cinerea*. *Fungal Genet Biol*, **47**, 917-921.
9. Muraguchi, H. and Kamada, T. (2000) A mutation in the gene encoding a cytochrome P450 of *Coprinus cinereus* affects mushroom morphogenesis. *Fungal Genet Biol*, **29**, 49-59.
10. Arima, T., Yamamoto, M., Hirata, A., Kawano, S. and Kamada, T. (2004) The *eln3* gene involved in fruiting body morphogenesis of *Coprinus cinereus* encodes a putative membrane protein with a general glycosyltransferase domain. *Fungal Genet Biol*, **41**, 805-812.
11. Muraguchi, H., Fujita, T., Kishibe, Y., Konno, K., Ueda, N., Nakahori, K., Yanagi, S.O. and Kamada, T. (2008) The exp1 gene essential for pileus expansion and autolysis of the inky cap mushroom *Coprinopsis cinerea* (*Coprinus cinereus*) encodes an HMG protein. *Fungal Genet Biol*, **45**, 890-896.
12. Plaza, D.F., Lin, C.W., van der Velden, N.S., Aebi, M. and Kunzler, M. (2014) Comparative transcriptomics of the model mushroom *Coprinopsis cinerea* reveals

tissue-specific armories and a conserved circuitry for sexual development. *BMC Genomics*, **15**, 492.
13. Muraguchi, H. and Kamada, T. (1998) The ich1 gene of the mushroom *Coprinus cinereus* is essential for pileus formation in fruiting. *Development*, **125**, 3133-3141.
14. Navarro González, M. (2008), Georg-August-Universität Göttingen, Göttingen, German.
15. Murata, Y., Fujii, M., Zolan, M.E. and Kamada, T. (1998) Molecular analysis of pcc1, a gene that leads to A-regulated sexual morphogenesis in *Coprinus cinereus*. *Genetics*, **149**, 1753-1761.
16. Lohse, M., Drechsel, O., Kahlau, S. and Bock, R. (2013) Organellar GenomeDRAW - a suite of tools for generating physical maps of plastid and mitochondrial genomes and visualizing expression data sets. *Nucleic Acids Res*, **41**, W575-581.
17. Kumar, S., Stecher, G. and Tamura, K. (2016) MEGA7: Molecular evolutionary genetics analysis version 7.0 for bigger datasets. *Mol Biol Evol*, **33**, 1870-1874.
